# CRISPR activation screen identifies TGFβ-associated PEG10 as a crucial tumor suppressor in Ewing sarcoma

**DOI:** 10.1038/s41598-022-12659-7

**Published:** 2022-06-23

**Authors:** Vadim Saratov, Quy A. Ngo, Gloria Pedot, Semjon Sidorov, Marco Wachtel, Felix K. Niggli, Beat W. Schäfer

**Affiliations:** 1 Department of Oncology and Children’s Research Center, University Children’s Hospital, Steinwiesstrasse 32, 8032 Zurich, Switzerland; 2 Experimental Infectious Diseases and Cancer Research, Children’s Research Center, University Children’s Hospital of Zurich, University of Zurich, Zurich, Switzerland

**Keywords:** Cancer, Oncogenes, Paediatric cancer, Sarcoma

## Abstract

As the second most common pediatric bone and soft tissue tumor, Ewing sarcoma (ES) is an aggressive disease with a pathognomonic chromosomal translocation t(11;22) resulting in expression of EWS-FLI1, an “undruggable” fusion protein acting as transcriptional modulator. EWS-FLI1 rewires the protein expression in cancer cells by activating and repressing a multitude of genes. The role and contribution of most repressed genes remains unknown to date. To address this, we established a CRISPR activation system in clonal SKNMC cell lines and interrogated a custom focused library covering 871 genes repressed by EWS-FLI1. Among the hits several members of the TGFβ pathway were identified, where PEG10 emerged as prime candidate due to its strong antiproliferative effect. Mechanistic investigations revealed that PEG10 overexpression caused cellular dropout via induction of cell death. Furthermore, non-canonical TGFβ pathways such as RAF/MEK/ERK, MKK/JNK, MKK/P38, known to lead to apoptosis or autophagy, were highly activated upon PEG10 overexpression. Our study sheds new light onto the contribution of TGFβ signalling pathway repression to ES tumorigenesis and suggest that its re-activation might constitute a novel therapeutic strategy.

## Introduction

Ewing sarcoma (ES) is an aggressive form of pediatric bone tumor^1,2^. The 5-year survival rate is close to 70%, dropping to 20–30% in patients with recurrent, refractory, or metastatic disease^3^. Currently, ES is treated with intensive multiagent chemotherapy, surgery, and high-dose radiation therapy. However, this therapy has severe side effects, is non-effective in disseminated cases and thus addition of targeted therapies is urgently needed.

The pathognomonic genetic aberration in ES fuses the EWS gene (also known as *EWSR1*, ES breakpoint region 1) to one of five erythroblast transformation-specific (ETS) transcription factors (TFs). Friend leukemia virus integration 1 (*FLI1*) is the fusion partner in approx. 85% of cases, v-ets erythroblastosis virus E26 oncogene homolog (*ERG*) in only 10% and others in less than 1%^4^.

The most common EWS-ETS fusion, EWS-FLI1, results from a t(11;22)(q24;q12) reciprocal translocation that combines the N-terminal EWS transactivation domain with the C-terminal ETS DNA-binding domain of FLI1^1^. EWS-FLI1 is an aberrant transcription factor, which is crucial for ES development, because of its ability to regulate the expression of a large number of target genes and thus orchestrate the oncogenic process underlying malignant transformation as of today unknown progenitor cells. This suggests that target genes offer promising opportunities to develop novel targeted therapies, especially since EWS-FLI1 itself as a transcription factor is still widely considered “undruggable” ^5^.

Several studies have been conducted in the past years to identify EWS-FLI1 target genes that play decisive roles in ES pathogenesis and maintenance. As a result, many genes have been identified and revealed some key pathways relevant for Ewing pathogenesis^6^. Among the upregulated and activated genes are for example insulin-like growth factor (IGF) binding protein 3 and the IGF pathway^7^, Aurora A and B kinases involved in cell cycle regulation^8^, and GLI1, a component of the canonical Hedgehog pathway^9–11^. In contrast, the contribution of repressed target genes is largely unknown. Some of the few available studies suggest that transforming growth factor-β receptor II (TGFBR2) promoter activity is suppressed directly by EWS-FLI1^12–15^ and restoration of TGFBR2 expression in Ewing cell lines can render them sensitive to TGFβ and thereby block tumorigenicity^12,16,17^.

Despite these studies, for the vast number of EWS-FLI1 target genes, their function in the tumorigenic process remains unknown while their characterization could potentially lead to development of less toxic and more specific therapies. Furthermore, most studies have investigated the impact of target genes one gene at a time, and a comprehensive comparison of the importance of target genes for maintenance and survival of tumor cells has not been conducted yet. This comparison is essential for the identification of pathways that are driving tumorigenesis as opposed to passenger pathways since dysregulations orchestrated by EWS-FLI1 are complex.

Due to technical advances in the field of high-throughput screens employing CRISPR-Cas9 systems, which allow efficient gene perturbation and even activation at the whole-genome scale^18–20^, it is now possible to address this question in a more comprehensive way. Specifically, introduction of modified Cas9 allows to modulate transcription without modifying the genomic sequence via fusing inactive or “dead” Cas9 (dCas9) to transcriptional activation or repression domains like VP64 or KRAB, respectively^20–22^.

Here, we generated a custom CRISPR activation (CRISPRa) library that we called LIBerty, targeting 871 genes repressed by EWS-FLI1, based on published studies, and introduced it to a clonal SKNMC cell line homogenously expressing the synergistic activation mediator (SAM) CRISPRa system to functionally interrogate repressed EWS-FLI1 target genes. Here we report that our screen identified several genes involved in the non-canonical TGFβ pathway that negatively affected viability of ES cells.

## Results

### Dropout screen with a custom CRISPRa library identifies several members of the non-canonical TGFβ pathway

**Figure 1:**
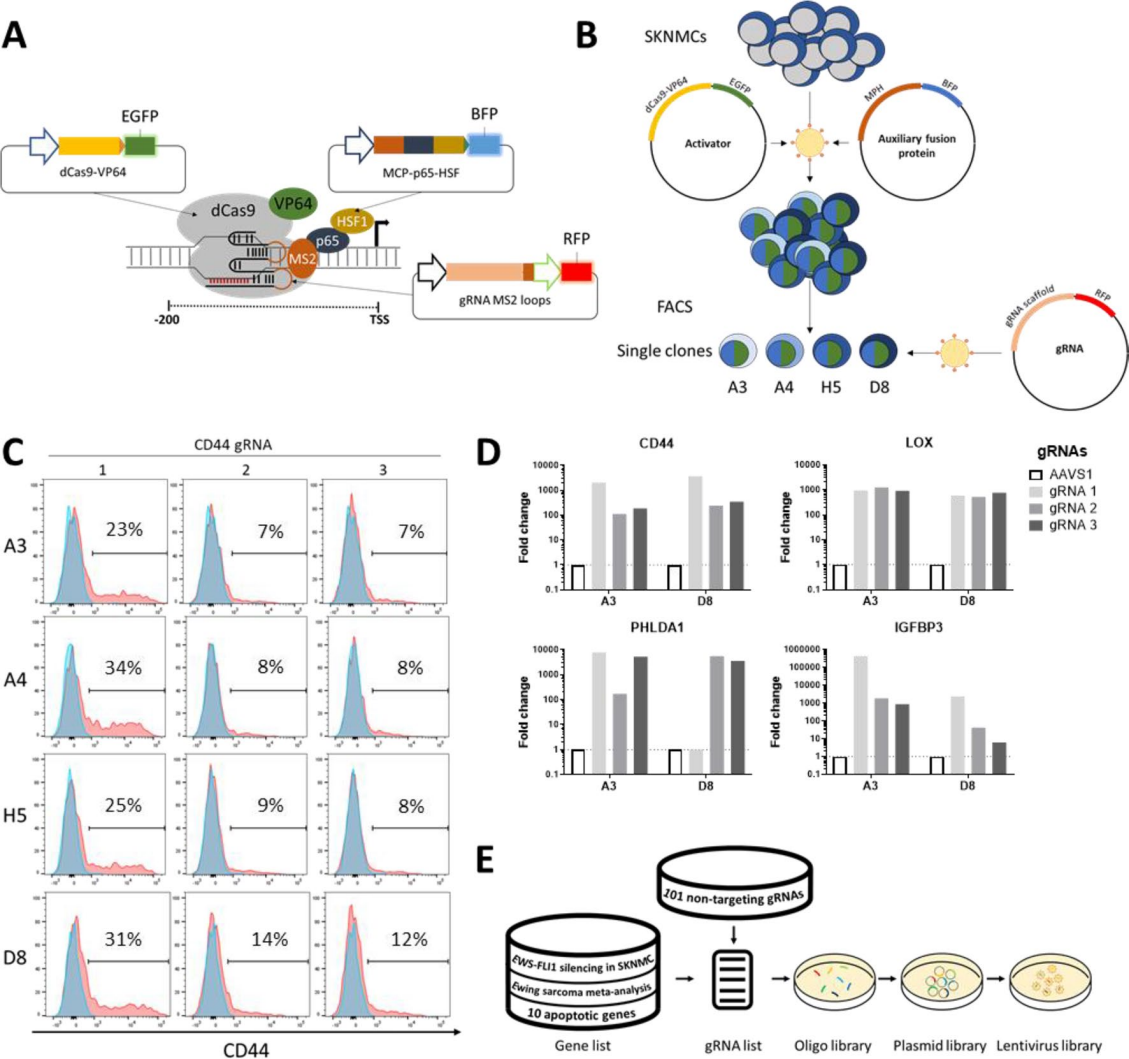
Establishment of the CRISPRa cell line. (**A**) Schematic representation of the modified CRISPRa Synergistic Activation Mediator (SAM) system (Based on 22). (**B**) Schematic representation of the clonal cell line generation. (**C**) Expression of the surface marker CD44 was measured by flow cytometry after introduction of CD44 targeting gRNAs. The percentage of positive cells for three gRNAs and four cell clones (A3, A4, H5 and D8) is indicated. Image was created by using FlowJo version 10, https://www.flowjo.com. (**D**) mRNA expression of four repressed genes (CD44, LOX, PHLDA1, and IGFBP3) was measured by qRT-PCR after gRNA transduction in the clones A3 and D8. Data is indicated as fold change over non-targeting control guide AAVS1, normalized to GAPDH. Image was created by using GraphPad version 8, https://www.graphpad.com. (**E**) Schematic representation of the gene selection for the lentiviral library LIBerty.

A CRISPRa system was established in SKNMC cells, a commonly used ES cell line. Among available CRISPRa systems, the Synergistic Activation Mediator (SAM) system^23^ was chosen based on its superior performance compared to other CRISPRa systems^24^. The original system was modified to feature fluorophore markers instead of antibiotics resistances (Fig. 1A).

SKNMC cells were transduced lentivirally with plasmids expressing dCas9-VP64 and MCP-p65-HSF1 and subsequently single-cell sorted via flow cytometry (Fig. 1B). The clones were assessed and selected based on homogeneity and efficiency of activation. To this end, CD44 as a marker for mesenchymal stem cells^25^ and being absent on the surface of SKNMCs was interrogated. CD44 overexpression was induced by transduction of plasmids containing three different gRNAs. The percentage of CD44 expressing cells in the clones increased from 7 to 12% to 23–34% as measured by flow cytometry (Fig. 1C). Furthermore, two clones (A3 and D8) were validated by introduction of guides targeting the known repressed EWS-FLI1 target genes LOX, PHLDA1, and IGFBP3^6,26^. RNA expression of these genes was induced by 10 to more than 1000 fold compared to GAPDH control (Fig. 1D). Because of its superior CD44 overexpression, we chose the clonal cell line SKNMC SAM D8 as basis for the screen.

A library of repressed EWS-FLI1 target genes was constructed based on published EWS-FLI1 knockout studies performed in SKNMC cells^27,28^. Genes with expression levels of at least 25% log2 fold higher in EWS-FLI1 knockout cells than in mock GFP knockout cells at 1% false discovery rate (FDR) were considered as repressed genes. Additionally, repressed genes were selected using the results of a meta-analysis across numerous Ewing cell lines and patient samples by Hancock et al.^29^. The resulting library consisted of 871 genes (Supplementary Table S1A) and included 101 non-targeting gRNAs as negative controls from another whole-genome library^20^, and 10 apoptotic effectors (BAD^30^, BAK1^31^, BAX^31^, BBC3 (PUMA)^32^, BCL2L11^33^, BID^34^, BOK^34^, HRK^35^, HUWE1^36^, PMAIP1^37^) as positive controls (Fig. 1E, Supplementary Fig. S1). The sequences for 3777 gRNAs, with at least three gRNAs per gene, were retrieved from the whole genome CRISPRa library developed by Zhang et al.^19^. The focused custom library was named “LIBerty” for its purpose of overcoming the repression by EWS-FLI1 (Supplementary Table S2A).

**Figure 2:**
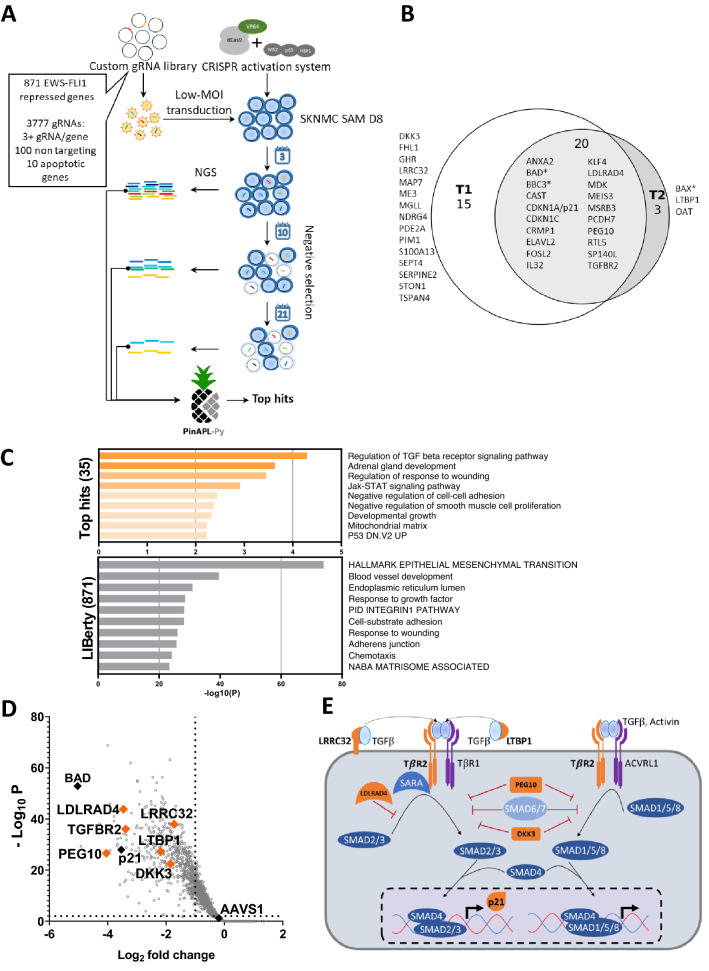
Top candidate genes identified by the CRISPRa dropout screen. **(A**) Schematic representation of the CRISPRa dropout screen. (**B**) List of 35 top-ranked genes grouped by timepoint (T1—10 days post transduction, T2—21 days post transduction). Genes marked with an asterisk are positive controls. 19 genes were identified at both time points (**C**) Metascape GO-term analysis of all 35 top candidates (upper panel) and the entire library (lower panel). Image was created by using GraphPad version 8, https://www.graphpad.com. (**D**) Result of the screen from time point 1. Each dot represents a single gRNA. Best performing gRNAs are labeled with the symbol of corresponding target gene. Controls are indicated in black, genes belonging to the TGFβ pathway in orange. Image was created by using GraphPad version 8, https://www.graphpad.com. (**E**) Schematic representation of the candidate gene hits in the TGFβ pathway indicated in orange.

Next, the LIBerty library was lentivirally delivered into SKNMC SAM D8 cells and a very high gRNA coverage of approximately 2500× was reached. Four biological replicates were sequenced at three, ten, and twenty-one days after transduction. The time point three days after transduction was considered as baseline, while ten days after transduction was the first and twenty-one days the second experimental time point (Fig. 2A). All samples were then sequenced by next generation sequencing (NGS). The NGS data were evaluated with the analysis tool PinAPL-Py^38^. Statistical comparison of the replicates for the QC showed very high correlation coefficients (Pearson > 0.973, Spearman > 0.982) (Supplementary Fig. S2). The non-targeting gRNAs showed, as expected, no or only a slight gRNA depletion (Supplementary Fig. S3). The analysis of the gRNA depletion was carried out using the adjusted robust rank aggregation (αRRA) method and a 5% FDR restriction. Hits with combined p-values across all replicates below 0.05 were considered significant, which resulted in thirty-five genes above the threshold for the first time point and twenty-three genes for the second time point (Supplementary Table S1B,C). Upon comparing the gene lists from both time points, twenty genes were identified to be in common. The combined top hit list for both time points was comprised of thirty-eight genes. Among them, three hits were the positive controls BAD, BBC3 and BAX (Fig. 2B). The remaining thirty-five candidates were further investigated for gene ontology (GO) term enrichment via the Metascape platform^39^. Importantly, the GO-term enrichment of the top hits (Fig. 2C, upper panel) did not overlap with the distribution in LIBerty (Fig. 2C, lower panel), confirming that the enriched pathways were likely to be true enrichments. Intriguingly, the most enriched GO-term was regulation of the TGFβ signaling pathway, which was represented by six genes from thirty-five top hits, namely TGFBR2, low density lipoprotein receptor class a domain containing 4 (LDLRAD4) , leucine rich repeat containing 32 (LRRC32), paternally expressed gene 10 (PEG10), latent transforming growth factor beta binding protein 1 (LTBP1), and dickkopf-3 (DKK3). To demonstrate the perfomance of the gRNAs targeting these genes, including BAD and p21 as positive controls and AAVS1 as negative control, gRNA read count fold change was plotted in a volcano plot (Fig. 2D). The gRNAs highlighted were one of the multiple gRNAs targeting a gene that caused the strongest dropout among those gRNAs. These most effective gRNAs will be further referred to as gRNA 1 (Supplementary Table S3). For example, best performing gRNA targeting TGFBR2 (TGFBR2 gRNA 1) led to a decrease of the read count of 10 and 20 fold compared to baseline for the first and second time points, respectively. Similarly, other gRNAs 1 showed reduction of read counts of more than threefold for the first time point and eightfold for the second time point (Supplementary Table S2B,C). AAVS1 derived from a sequence from the “safe harbor” locus^40^ and included as negative control in the library, expectedly only mildly affected cell proliferation and displayed a read count fold change of 0.9 compared to the baseline for the first time point and 0.6 for the second. On the other hand, gRNAs targeting BAD, an apoptotic effector^30^, as well as CDKN1A or p21, a negative regulator of the cell cycle, caused some of the strongest read count fold changes in the whole screen. Thus, BAD gRNA 1 ranked first and second among all gRNAs at first and second time points, respecitvely, by causing a dropout of over 96% of cells. CDKN1A gRNA 1 was in seventeenth place at both time points causing dropout of over 91% of cells. (Supplementary Table S2B,C). Based on this perfomance, BAD and CDKN1A gRNA 1 were used further as positive controls.

Among the hits, TGFBR2 can be found ranking eleventh at time point one and seventh at time point two (Supplementary Table S1B,C). TGFBR2 is a known directly repressed target of EWS-FLI1 that has an antitumoral effect upon overexpression in ES. LDLRAD4 is a suppressor of the canonical TGFβ signaling via binding to SMAD complexes and preventing signal propagation^41^. LRRC32 is suggested to bind TGFβ on the cell surface and where it acts as an anchor ^42^. PEG10 is known to be able to suppress the canonical TGFβ signaling^43^. LTBP1 regulates TGFβ signaling by binding TGFβ to the extracellular matrix creating deposits^44^. DKK3 is a tumor suppressor gene, known to be involved in the Wnt pathway, with an inhibitory effect on the canonical TGFβ signaling^45^. The presence of TGFBR2, BAD, and p21 among top hits (Fig. 2B,D) further confirms the validity of our screen. In summary, we identified the TGFβ signaling pathway as a primary pathway whose repression appears necessary for ES cell growth. Six genes were attributed to this pathway based on their possible interactions described in the literature (Fig. 2E) ^41–46^.

### PEG10 re-expression induces a strong anti-proliferative effect

**Figure 3:**
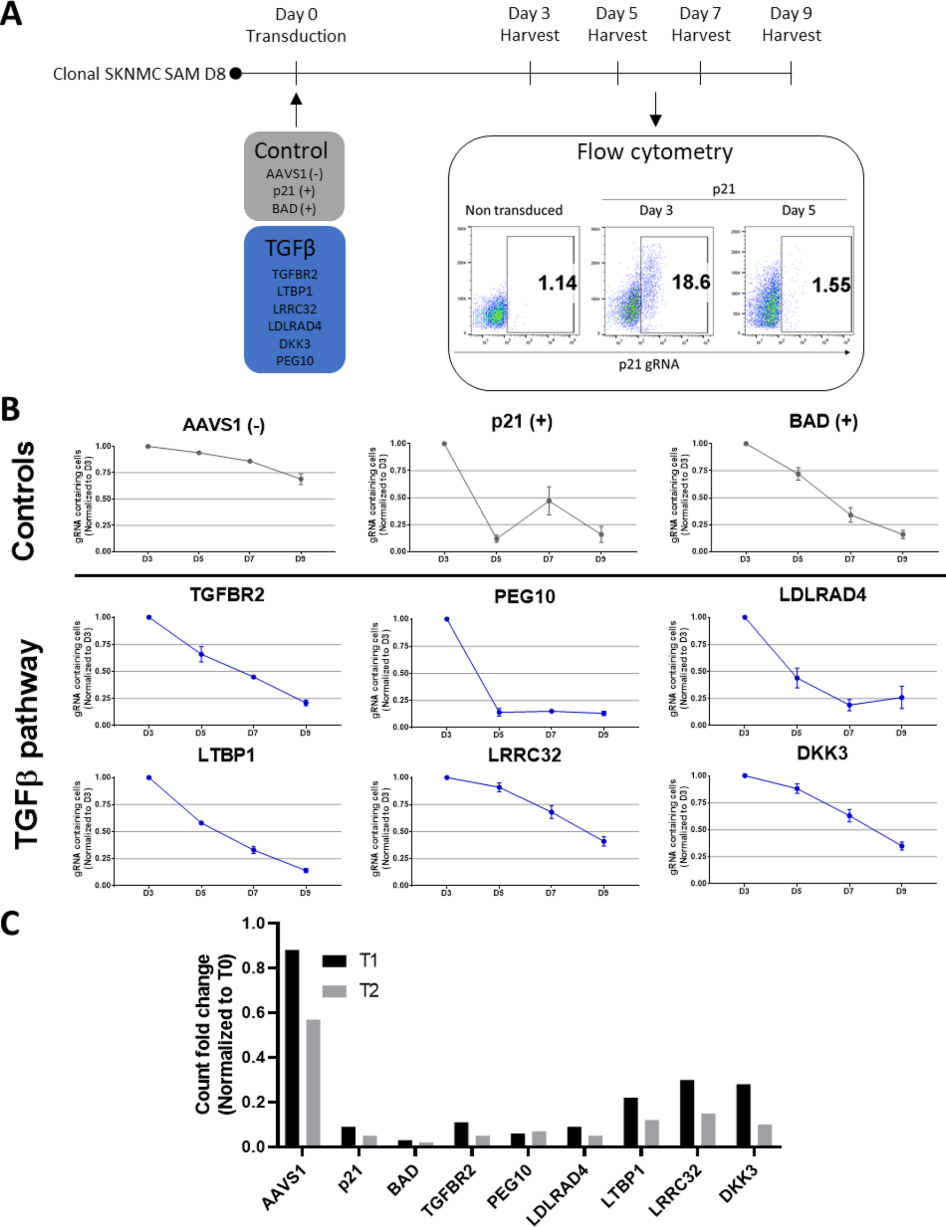
Competition assay confirms the antitumoral effect of the hits identified by the dropout screen. **(A**) Schematic representation of the competition assay in SKNMC SAM D8 clone. The clonal SKNMC SAM D8 cells were transduced with a set of control gRNAs (AAVS1 as negative control and p21 and BAD as positive controls) and a set of gRNAs targeting the genes of interest in the TGFβ pathway. The fraction of the cells expressing the gRNAs was measured via flow cytometry every second day for nine days with day three post transduction as baseline. In the example, the fraction of cells expressing p21 gRNA constitutes 18.6% of all cells on day three post transduction and is reduced to 1.5% on day five post transduction. Image was created by using FlowJo version 10, https://www.flowjo.com. (**B**) Validation of the top hits via competition assay. The ratio of the gRNA containing cells represents the size change of the cell population expressing the respective gRNA over nine days post transduction and is normalized to the size of the fraction on day three post transduction. Reduction of the ratio reflects a dropout of the cells expressing the gRNA and thus overexpressing the respective gene. Image was created by using GraphPad version 8, https://www.graphpad.com. (**C**) Fold change of read count from the CRISPR activation screen of the best performing gRNAs (gRNA 1) targeting the set of genes of interest in the TGFβ pathway at time point 1 (T1) and time point two (T2) compared to time point zero (T0). Image was created by using GraphPad version 8, https://www.graphpad.com.

To validate the above-mentioned six candidate genes from the screen, we chose the single gRNAs for each of these that exhibited the strongest dropout ratio. Cells from the D8 clone were lentivirally transduced with the chosen single gRNAs as well as negative (AAVS1) and positive controls (p21 and BAD) with various amounts of lentivirus with the aim to achieve transduction efficiency of 50% or below. The transduced cells are expected to overexpress the targeted gene and, in case of a proliferative disadvantage, these cells will be overgrown and outcompeted by non-transduced cells, whereby the population of transduced cells can be traced using the fluorescent marker included in the vector. Over a period of nine days starting from the third day after transduction, samples were collected every second day, and relative percentage of transduced cells was assessed via flow cytometry (Fig. 3A). While the number of cells with the negative control AAVS1 gRNA over nine days was reduced to 72%, that of cells overexpressing gRNA of all other six candidate genes decreased to 10—20%, on par with cells overexpressing gRNA of the two positive controls BAD and p21 (Fig. 3B). In particular, compared to all other candidates in the competition assay, PEG10 overexpression caused the fastest and strongest dropout, where the cell population was reduced to only 14% of the original population already at day five after transduction (Fig. 3B,C). Therefore, PEG10 was selected as a gene of interest for further investigation.

### PEG10 overexpression reduces cell numbers in different SKNMC clones

**Figure 4:**
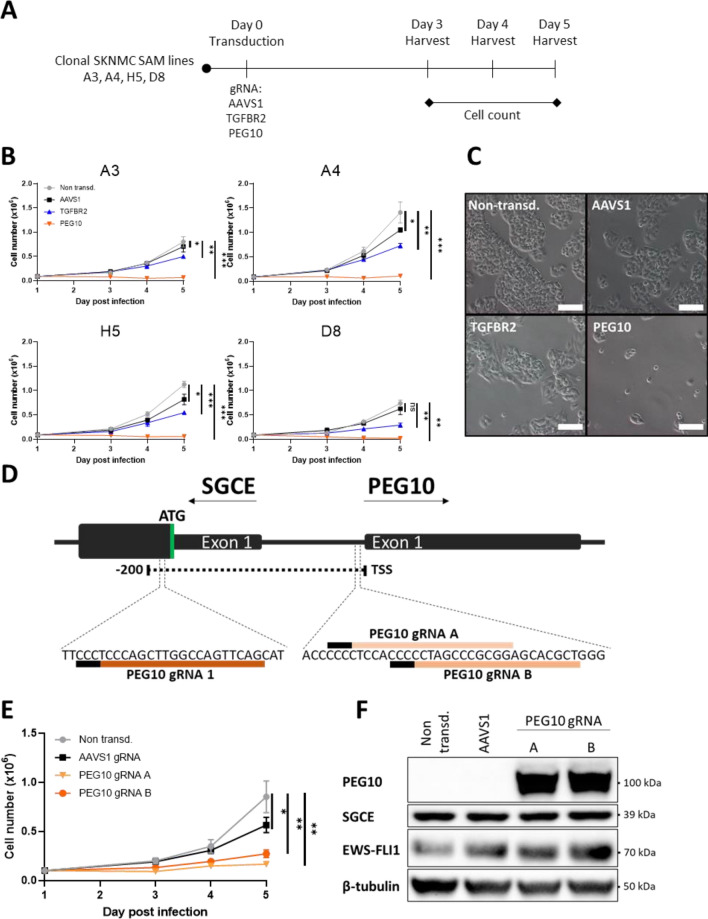
PEG10 overexpression in clonal SKNMC SAM cell lines reduces cell growth. **(A**) Schematic representation of the cell proliferation assay. Clonal cell lines SKNMC SAM A3, A4, H5, D8 were transduced with gRNAs directed towards AAVS1, TGFBR2 and PEG10 and counted on day three, four and five post transduction. (**B**) Quantification of the number of TGFBR2 and PEG10 overexpressing SKNMC SAM cells (n = 1,* p < 0.1, **p < 0.01, ***p < 0.001, paired t-test). Image was created by using GraphPad version 8, https://www.graphpad.com. (**C**) Representative image of the SKNMC SAM D8 cell line on day 5 post transduction overexpressing TGFBR2 and PEG10. 100 μm scale bar. (**D**) Schematic representation of the genomic locus around the transcription start sites (TSS) of SGCE and PEG10 including the two additional gRNA sequences of PEG10 gRNAs and the frame of 200 base pairs for efficient placement of gRNAs for CRISPRa. (**E**) Quantification of the number of SKNMC SAM D8 cells overexpressing PEG10 via PEG10 gRNAs that were not used in the screen (n = 1,* p < 0.1, **p < 0.01, ***p < 0.001, paired t-test). Image was created by using GraphPad version 8, https://www.graphpad.com. (**F**) PEG10, SGCE and EWS-FLI1 protein levels in SKNMC SAM D8 cells overexpressing PEG10. Whole cell lysates for Western Blot harvested on day 5 post transduction. Image was created by using Image Lab version 6.1, https://www.bio-rad.com/en-ch/product/image-lab-software.

PEG10 is a highly conserved imprinted gene in mammals and research in mice implies an important role in placenta formation and adipocyte differentiation^47^. Interestingly, PEG10 has also been shown to interact with the TGFβ receptor family^48^. To further validate the physiological effect of PEG10 overexpression in different CRISPRa clones, we lentivirally transduced each of four SKNMC SAM clones (A3, A4, H5, and D8) with a single gRNA to induce PEG10 expression, AAVS1 gRNA 1 as negative control or TGFBR2 gRNA 1 as positive control. Cell numbers were determined on days three, four, and five after transduction via an automatic cell counter (Fig. 4A). AAVS1 gRNA containing cells grew between seven- and 11-fold whereas cell number of PEG10 gRNA containing cells declined below the seeded amount already on day three post infection (Fig. 4B and Supplementary Fig. S4). This suggests that PEG10 overexpression exhibits a detrimental effect on cell numbers in all clonal cell lines, a finding which was also obvious upon morphological examination (Fig. 4C).

Interestingly, the targeting window for CRISPRa gRNAs to activate PEG10 overlaps with the transcriptional start site of Sarcoglycan Epsilon (SGCE), which is an imprinted gene encoding for ε-sarcoglycan located on the reverse DNA strand (Fig. 4D). SGCE expression therefore could also have been affected by the CRISPRa machinery, thus being co-responsible for the observed physiological effects (Fig. 4B,C). Therefore, two additional gRNA sequences targeting PEG10 and being further away from SGCE were generated using the CRISPick platform^49,50^ to verify that the antiproliferative effect originated exclusively from PEG10 overexpression and not from off-target effects. These gRNAs were lentivirally transduced into the SKNMC SAM D8 cell line and cell numbers were again monitored over five days. Both gRNAs induced overexpression of PEG10 and reduction of cell numbers throughout the experiment (Fig. 4E,F). Furthermore, no change in SGCE protein levels was observed in under these conditions (Fig. 4F). Hence, the gRNAs targeting PEG10 do not exhibit off-target effects via overexpression of SGCE.

### PEG10 expression affects cell proliferation in a dose-dependent manner

**Figure 5:**
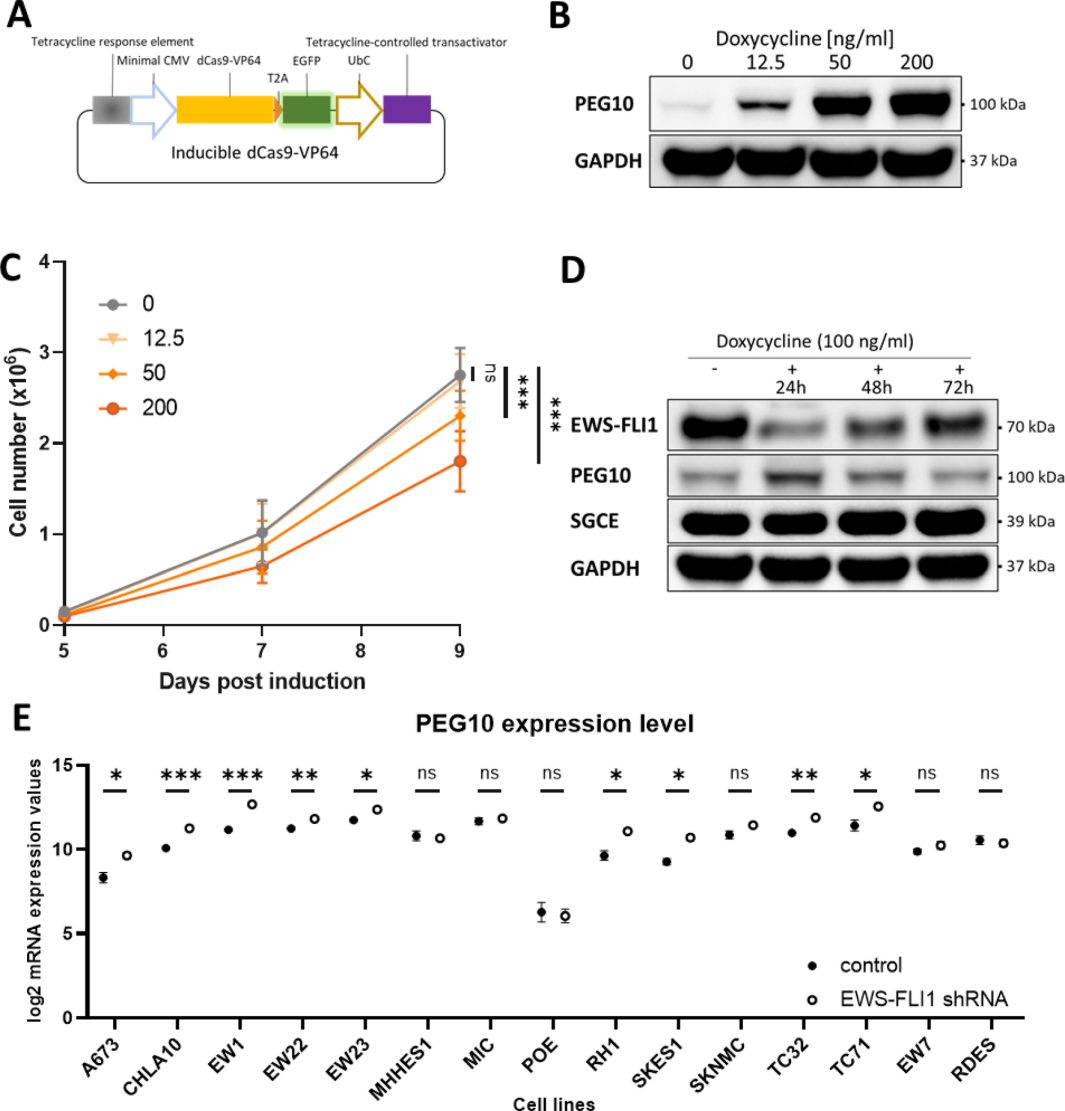
PEG10 expression affects cell proliferation in a dose-dependent manner and is repressed in the presence of EWS-FLI1. **(A**) Schematic representation of the inducible dCas9-VP64 construct. (**B**) PEG10 protein level in SKNMC cells with the inducible CRISPRa system overexpressing PEG10 that were harvested 4 days post induction. Image was created by using Image Lab version 6.1, https://www.bio-rad.com/en-ch/product/image-lab-software. (**C**) Quantification of the number of SKNMC cells with the inducible CRISPRa system overexpressing PEG10 over 9 days post induction (n = 2,* p < 0.1, **p < 0.01, ***p < 0.001, paired t-test). Image was created by using GraphPad version 8, https://www.graphpad.com. (**D**) EWS-FLI1, PEG10 and SGCE protein levels in SKNMC cells with the inducible EWS-FLI1 shRNA construct upon induction for 24, 48 and 72 h. Data is representative for 3 biological replicates. Image was created by using Image Lab version 6.1, https://www.bio-rad.com/en-ch/product/image-lab-software. (**E**) PEG10 mRNA expression levels across 15 EWS-FLI1 positive Ewing sarcoma cell lines after EWS-FLI knockdown via inducible EWS-FLI1 shRNA for 96 h. Data was extracted from the Ewing Sarcoma Cell Line Atlas (51) (n = 3,* p < 0.1, **p < 0.01, ***p < 0.001, paired t-test). Image was created by using GraphPad version 8, https://www.graphpad.com.

To investigate whether the level of PEG10 expression influences regulation of cellular proliferation, we generated a plasmid containing a doxycycline-inducible dCas9-VP64 construct and GFP as fluorophore marker (Fig. 5A). SKNMC cells were lentivirally transduced with plasmids containing this construct together with MCP-p65-HSF1 and PEG10 gRNA 1. Transduced cells were sorted to enrich for cells containing all three constructs. In these, expression of PEG10 was then induced with increasing concentrations of doxycycline and cell numbers determined every second day for a total of nine days. The performance of the inducible CRISPRa system was evaluated on day three after induction and exhibited discernible and dose-dependent expression of PEG10 upon induction with doxycycline (Fig. 5B). A difference in cell numbers became apparent after day five of induction. While the lowest concentration (12.5 ng/ml) of doxycycline did not reduce proliferation compared to non-induced cells, treatment with the two higher doxycycline concentrations (50 and 200 ng/ml) lead to a dose-dependent reduction of cell numbers (Fig. 5C). This result suggests that a certain threshold of PEG10 protein levels is required to reduce ES cell proliferation. Next, we confirmed that EWS-FLI1 is repressing PEG10 expression in SKNMC cells. A previously established SKNMC cell line containing shRNA targeting EWS-FLI1 was used^51^. The shRNA expression was induced with doxycycline (100 ng/ml) and whole cell lysate for Western Blot was harvested 24, 48, and 72 h after induction (Fig. 5D). At 24 h after induction, silencing of EWS-FLI1 was evident as the protein level was reduced to circa 17% compared to non-induced controls. An increase in the PEG10 protein level to 209% as compared to the control was also apparent. At the next time points of 48 and 72 h, the level of EWS-FLI1 returned closer to levels in non-induced cells with 43% and 58%, of the basal level, respectively. Inversely, the increased levels of PEG10 with respect to the non-induced level was down to 203% and 137% at 48 and 72 h, respectively (see densitometry analysis and replicates in Supplementary Fig. S5). Again, SGCE levels remained mostly unchanged throughout the whole experiment, suggesting that EWS-FLI1 does not influence the expression of SGCE.

The Ewing Sarcoma Cell Line Atlas (ESCLA) is a recently published database containing data from 15 Ewing cell lines with inducible EWS-FLI1 knockdown that have been profiled by various methods including gene expression arrays^52^. The available expression data for PEG10 were extracted upon induction of the EWS-FLI1 knockdown for 96 h (Fig. 5E). Indeed, 9 out of 15 cell lines exhibited significantly higher mRNA levels of PEG10 upon EWS-FLI1 knockdown independent of the knockdown efficiency, suggesting that EWS-FLI1 represses expression of PEG10 across a majority of Ewing cell lines.

### PEG10 overexpression causes cell death via non-canonical TGFβ pathways

**Figure 6:**
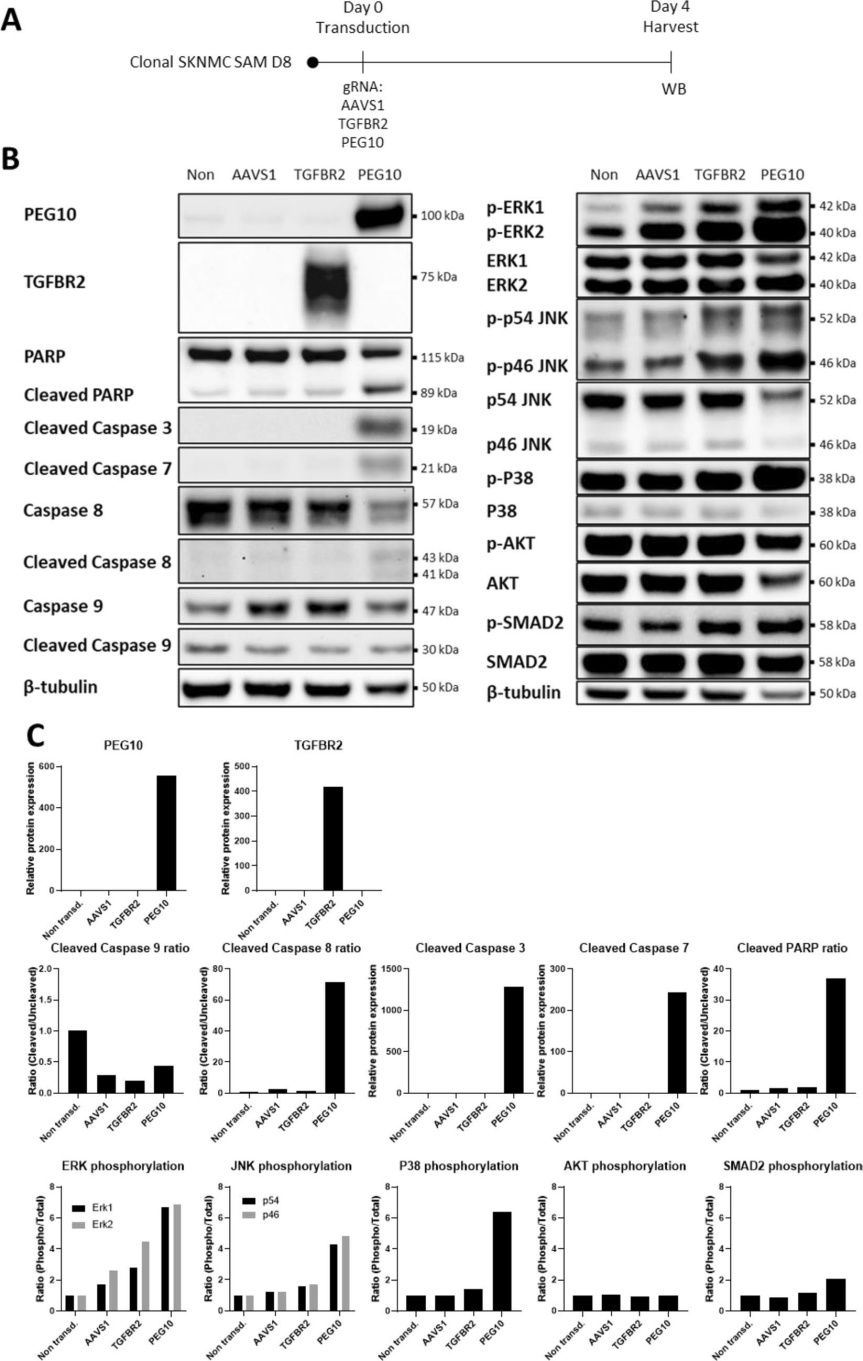
Overexpression of PEG10 in SKNMC SAM D8 cells leads to detectable levels of apoptosis potentially via activation of non-canonical TGFβ pathways. **(A**) Schematic representation of the cell proliferation assay. (**B**) SKNMC SAM D8 cells overexpressing TGFBR2 and PEG10 were harvested on day 4 post transduction. The whole cell lysate was analyzed using a panel of antibodies as indicated with β-tubulin as control. TGFBR2 and PEG10 antibodies were used to confirm overexpression of the proteins. PAPR, cleaved caspase 3, 7, 8 and 9 were used as apoptosis markers (left panel). Phosphorylation levels of ERK1/2, SAPK/JNK, P38, AKT, SMAD2 were monitored to measure the activity of the different non-canonical pathways downstream of TGFβ (right panel). The panel is representative for 3 biological replicates. Image was created by using Image Lab version 6.1, https://www.bio-rad.com/en-ch/product/image-lab-software. (**C**) Densitometry of the Western Blot measuring PEG10, TGFBR2 protein levels, cleavage status of PARP, Caspase 3, 7, 8 and 9, phosphorylation status of ERK1/2, SAPK/JNK, P38, AKT and SMAD2 in TGFBR2 and PEG10 overexpressing SKNMC SAM cells 4 days post transduction. β-tubulin was used to normalize relative protein expression of PEG10, TGFBR2, Caspase 3 and 7. Cleaved PARP, Caspase 8 and 9 were normalized with total length PARP, Caspase 8 and 9. Phosphorylated ERK1/2, SAPK/JNK, P38, AKT and SMAD2 were normalized with total ERK1/2, SAPK/JNK, P38, AKT and SMAD2. Image was created by using GraphPad version 8, https://www.graphpad.com.

To explore the mechanisms behind the antiproliferative effect of PEG10, we transduced SKNMC SAM D8 cells with gRNAs targeting AAVS1 (negative control), TGFBR2 (positive control), and PEG10. Whole cell lysates for Western Blot were obtained four days after transduction (Fig. 6A). The gRNAs targeting TGFBR2 and PEG10 facilitated high overexpression of the respective target genes. Furthermore, cells overexpressing PEG10 underwent Caspase 8-initiated cell death as demonstrated by upregulation of apoptotic markers such as cleaved PARP and cleaved Caspases 3, 7 and 8 whereas cleavage status of Caspase 9 remained similar across all samples. The TGFβ pathway downstream of its receptor is comprised of a canonical and different non-canonical signaling cascades. The canonical pathway propagates the signal via SMAD proteins with the first protein in the cascade being SMAD2^53^. The most prominent among the non-canonical pathways are the RAF/MEK/ERK, MKK/JNK, MKK/P38, and PI3K/AKT axis^54^. Interestingly, PEG10 overexpressing cells showed a high induction of JNK, ERK, and P38 phosphorylation, whereas AKT phosphorylation remained constant (Fig. 6B,C, see densitometry and replicates in Supplementary Fig. S6). None of the extracts exhibited higher SMAD2 phosphorylation levels. These results suggest that the reduction of cell numbers in PEG10 overexpressing cells might be caused by activation of one or several non-canonical TGFβ pathways, ultimately leading to cell death.

## Discussion

ES is an aggressive tumor of the bone and soft tissues. Many genes have been identified to be part of pathways relevant for ES pathogenesis. However, a vast number of EWS-FLI1 target genes remains unexplored and their characterization could potentially lead to the development of much needed, less toxic, and more specific therapies.

Repressed genes most likely play similarly important roles in the tumorigenic process as overexpressed genes. Studies that allow investigation of genes that are normally repressed in tumor cells are still quite rare and have not been conducted in ES to date. Technically, such studies are now possible employing the so-called CRISPRa strategies using an enzymatically dead CRISPR enzyme (dCAS9) coupled to a transcriptional activation domain and guided to promoter regions of selected genes by gRNAs. In this study, we successfully generated a custom CRISPRa library “LIBerty” targeting genes repressed by EWS-FLI1 and introduced it to a clonal SKNMC cell line containing the CRISPRa system SAM. The dropout screen identified 35 hits of which we were able to put six (TGFBR2, LDLRAD4, LRRC32, PEG10, LTBP1, and DKK3) in the context of the TGFβ signaling pathway. This pathway is known for its major function as tumor suppressor^46^. Furthermore, TGFBR2, one of the top hits in the screen, is a known target of not only EWS-FLI1^12^ but also EWS-ERG and EWS-ETV1, two other fusion oncoproteins expressed by Ewing family tumors^13^. These findings underline the importance of repression of the TGFβ pathway.

Competition assay with single best performing gRNAs used in the screen confirmed the hits and allowed selection among six top hits. PEG10, one of the short-listed candidates, exhibited the strongest cell dropout effect and thus was chosen for further investigation. PEG10 is an imprinted retrotransposon-derived gene encoding for a protein acting as a transcription factor during brain development^55–57^. In addition to an essential role in placental development, the fact that PEG10 is highly conserved in eutherian mammalians suggests possible other crucial functions^58,59^. Aberrant PEG10 expression is linked to several human tumors such as neuroendocrine prostate cancer^60^ and hepatocellular carcinoma^61^. However, PEG10 has not been associated with ES before. Interestingly, PEG10 has been shown to interact with members of the TGFβ receptor family and inhibit downstream signaling^43,48,62^.

We confirmed the accuracy of our screen with multiple controls and additional readouts. We observed cell number reduction facilitated by PEG10 overexpression across several clonal SKNMC cell lines confirming that the observed effect was not due to a clonal effect. Further, using PEG10 gRNAs from another library, we verified that the antiproliferative effect was indeed not due to gRNA off-target effects. We also showed that upon PEG10 overexpression SGCE protein levels remained unaffected, confirming that SGCE is not involved in the observed phenotype. These results suggest that PEG10 is a true hit, independent of the possible clonal effect of the cell line, the gRNA off-target effect, or SGCE expression.

We also noted that PEG10 affects cell proliferation in a dose-dependent manner and that high PEG10 protein levels are necessary to cause cell number reduction. Probably, the cell reduction effect we observed is a drastic response to extremely high PEG10 levels. However, PEG10 expression is certainly detrimental to ES cancer cells. This is strengthened not only by results from our work but also by ESCLA data that suggest that PEG10 is indeed a target of EWS-FLI1 repression. Further studies are needed to determine whether PEG10 is a direct or an indirect target of EWS-FLI1.

Lastly, we investigated the mechanisms by which PEG10 activity reduces proliferation. We assessed canonical SMAD2 phosphorylation as well as several non-canonical or non-SMAD pathways including PI3K-Akt, JNK, p38, and Erk. The activated status of JNK, p38, and Erk but not SMAD2 and Akt suggests involvement of PEG10 in driving the signaling in ES cells towards certain non-canonical pathways. Caspase 8 and 9 are considered to be initiator caspases for the extrinsic and intrinsic apoptotic pathway, respectively^63^. Our data showed that Caspase 8 was cleaved only in cells overexpressing PEG10 whereas Caspase 9 cleavage status remained similar across all cells. Typically Caspase 8 is activated via death receptors. However, it has been shown that Caspase 8 can be activated independently via, for instance, TGFβ signaling^64^ or other signaling pathways such as p38, JNK^65^ and ERK^66^ which we have shown were activated upon PEG10 expression. Thus, these data suggest that the main mechanism of cell death observed in PEG10 overexpressing cells might be apoptosis induced by p38, JNK and ERK via Caspase 8 cleavage, however other forms of cell death such as autophay cannot be excluded currently. Potentially, PEG10 could influence the signaling at the TGFβ receptor complex as PEG10 has been shown to be able to bind to several members of the TGFβ receptor family^48^. There, PEG10 could possibly induce conformational changes in the TGFβ receptor complex steering the signaling cascade in direction of JNK, p38, and Erk activation. There is no direct link between PEG10 and non-canonical TGFβ pathway known yet. To confirm it, the interaction of PEG10 with the TGFβ receptor family will need to be studied in greater detail. Understanding this interaction might provide means to manipulate the TGFβ pathway to facilitate a strong antitumoral effect. A direct activation of the TGFβ pathway in ES might seem like a promising approach, however the pathway is highly complex and has a potent anti-proiferative effect in normal cells^67^. Clinically, such an approach therefore would require cell-specific activation which is quite challenging. Additional investigation of the specific role of PEG10 and its interaction with TGFβ could be carried out in a healthy cell closely related to ES such as mesenchymal stem cells. Overexpression of PEG10 and TGFBR2 in MSCs would help to shed light on their effect on cell proliferation and whether this effect is cancer-specific. Furthermore, more studies are needed to understand the contributions of each of the three downstream pathways to the induction of cell death, which might offer better insights into antitumoral pathways.

Alongside PEG10, our screen uncovered several additional genes outside the TGFβ pathway negatively affecting the proliferation of ES cells. For instance, IL32 has been shown to inhibit JAK/STAT signaling by interfering with the activation of STAT3^68^ and FHL1 or SLIM1 has been classified as a negative regulator of STAT signaling via ubiquitination of STATs^69^. These candidates might have tumor suppressor qualities and could be able to impede cell division and induce programmed cell death. All candidates are unique given the rarity of gene overexpression studies in ES and are certainly worth further investigation.

In summary, our study identified PEG10 as a repressed EWS-FLI1 target gene with a physiologically important antiproliferative effect. Investigation of the mechanisms behind this effect might provide new approaches for targeted therapy of ES.

## Material and methods

### Cell lines

All SKNMC cell lines were cultured in RPMI1640 (Sigma Aldrich) with 10% FBS (Sigma Aldrich), 2 mM GlutaMAX (ThermoFisher Scientific) and 100 U/ml penicillin/streptomycin (ThermoFisher Scientific) at 37 °C in 5% CO_2_. HEK293T cells were cultured in DMEM (Sigma Aldrich) with 10% FBS (Sigma Aldrich), 2 mM GlutaMAX (ThermoFisher Scientific) and 100 U/ml penicillin/streptomycin (ThermoFisher Scientific) at 37 °C in 5% CO_2_. The cells were split using either trypsin–EDTA (Sigma Aldrich) or Accutase (Sigma Aldrich). The cells were regularly tested for mycoplasma contamination with the MycoAlert (Lonza) and LookOut (Sigma Aldrich) mycoplasma detection kits.

### Reagents and antibodies

Doxycycline (Sigma Aldrich) was used for doxycycline-mediated induction. All used antibodies are summed up in the Supplementary Table S4.

### Plasmids and cloning

The dCAS9-VP64_GFP (Plasmid #61422), EF1a-MS2-p65-HSF1-2A-Hygro-WPRE (Plasmid #89308), lenti sgRNA(MS2)_puro optimized backbone (Plasmid #73797) and pHAGE TRE dCas9-VP64 (Plasmid #50916) plasmids were purchased from Addgene. In the MCP-p65-HSF1 construct hygromycin resistance was replaced with TagBFP from Lenti-Cas9-gRNA-TagBFP2 (Plasmid #124774) via NEBuilder HiFi DNA Assembly kit (New England Biolabs). In the lenti sgRNA(MS2)_puro optimized backbone puro resistance was replaced with TagRFP657 from sg_shuttle_RFP657 (Plasmid #134968) via NEBuilder HiFi DNA Assembly kit (New England Biolabs). The plasmid pHAGE TRE dCas9-VP64 was modified by replacement of dCas-VP64 with dCas9-VP64 from dCAS9-VP64_GFP (Plasmid #61422), removal of the IRES-Kanamycin fragment and addition of P2A-EGFP from dCAS9-VP64_GFP (Plasmid #61422) after dCas9-VP64 via NEBuilder HiFi DNA Assembly kit (New England Biolabs).

### Retrovirus production and transduction

The HEK293T cell were seeded with 80% confluency. The plasmids containing Pax2, VSVG and viral construct were mixed in a ratio of 1:0.7:1.7 and added to an 8.3 fold volume of an 1 mg/mL polyethyleneimine (PEI) stock solution (Sigma Aldrich). The whole mixture was then added dropwise to the cells. The viral supernatant was harvested 30 h post transfection, concentrated via Amicon^®^ Ultra-15 Centrifugal Filter Unit (Merck) and aliquoted for storage at −80 °C. Target cells were transduced by seeding cells with 50% confluency and incubating them with medium containing the viral supernatant for 24 h.

### Generation of new cell lines

The SKNMC SAM cell lines were generated by transduction of SKNMC cells with the dCas9-VP64 and MPH lentivirally packaged constructs with a multiplicity of infection (MOI) of approximately 0.3 and enriched via fluorescence-activated cell sorting (FACS) on the FACSAria Fusion (BD Biosciences) with the highest sorting purity (4 way purity setting) and single-cell sorting setup. SKNMC cells with inducible PEG10 expression were transduced with the inducible dCas9-VP64, MPH and PEG10 gRNA lentivirally packaged constructs with an MOI of approximately 0.3, induced with 100 ng/ml doxycycline and enriched via fluorescence-activated cell sorting (FACS) on the FACSAria II (BD Biosciences) with the highest sorting purity.

### Surface marker staining and flow cytometry

Circa 500.000 cells were stained with 0.25 μg of CD44 ApCAPC/Cy7 anti-mouse/human (BioLegend, #103028) in 25 μl volume in the dark and at 4 °C for 30 min. After washing with FACS buffer (PBS with 2% FBS (Sigma Aldrich)), the samples were acquired using a LSRFortessa flow cytometer (BD Biosciences) and the FACSDiva software. The data were analyzed with FlowJo version 10 (BD Biosciences).

### Quantitative RT-PCR

Total RNA extraction from SKNMC SAM D8 cells was done with the RNeasy kit (Qiagen) according to the manual of the manufacturer. Complementary DNA was synthesized with the High-Capacity Reverse Transcription Kit (ThermoFisher Scientific) according to the manual of the manufacturer. Quantitative RT-PCR was performed using TaqMan gene expression master mix and gene expression assays (ThermoFisher Scientific) targeting CD44 (Hs01075864_m1), LOX (Hs00942480_m1), PHLDA1 (Hs00378285_g1), IGFBP3 (Hs00365742_g1) and GAPDH (Hs02758991_g1). Cycle threshold (Ct) values were normalized to GAPDH and relative expression fold changes were calculated by the ΔΔCt method^70^.

### Custom gRNA library CRISPR activation screen

The LIBerty library has been assembled following closely the protocols described by Joung et al.^18^. A pooled library of oligomers was ordered from Twist Bioscience. The oligomers were amplified via PCR using specific primers (Supplementary Table S5) and NEBNext High Fidelity PCR Master Mix (New England Biolabs).

The gRNA MS2 scaffold containing plasmid was enzymatically digested and the plasmid library was constructed via Gibson assembly kit (New England Labs). The resulting gRNA library was purified and concentrated via isopropanol precipitation. The gRNA library was amplified in NEB-10 bacteria (New England Labs) after electroporation was carried out according to the manual. The amplified gRNA library was sequenced and passed the quality check suggested in the protocols (Supplementary Fig. S1).

The SKNMC SAM D8 cells were transduced with packaged gRNA lentiviral library at an MOI of approximately 0.3. Six 15 cm dishes with 25 × 10^6^ cells were incubated with viral supernatant for 24 h to establish correct gRNA coverage and cell number for harvesting. The cells were cultured in 15 cm dishes maintaining the gRNA coverage for the duration of the experiment. At each time point cells were detached with Accutase, washed with PBS and snap-frozen in liquid nitrogen for storage at -80 °C until DNA extraction was perfomed. For the extraction of the genomic DNA, the cells were thawed on ice and processed with the Blood & Cell Culture DNA maxi kit according to its manual. The gRNA sequences were amplified in a two-step PCR reaction. In the first step, a fragment of 854 bp was amplified using NEBNext High Fidelity PCR master mix (New England Biolabs). In the second step, 1% of the total product was used to amplify the gRNA sequence with the attached NGS adapters (Supplementary Table S5). The samples were size restricted via gel electrophoresis and quantified with the Qubit (ThemoFisher Scientific) before submission to the Functional Genomics Center Zurich where the samples where run on the Illumina NextSeq 500. The NGS data was analyzed on the platform PinAPL-Py version 2.8.1 with settings summed up in the Supplementary Data S1. The raw read count data calculated via PinAPL-Py as part of the analysis of the NGS data can be found in Supplementary Data S2. The resulting gene list was analyzed for GO-term enrichment using standard settings in the Metascape platform.

### Competition assay

The cells were seeded in six well plates (Sarstedt) and transduced with a three-step titration of the viral supernatant. The cells were then harvested for flow cytometry every second day starting three days after transduction. The cells were acquired measuring TagRFP657 signal corresponding to the signal of the reporter present in the gRNA construct. The samples were acquired with the LSRFortessa flow cytometer (BD Biosciences) and the FACSDiva software. The data were analyzed with FlowJo (BD Biosciences). Further analysis was carried out in Prism GraphPad version 8 by comparison of the size changes in TagRFP657 positive population over the course of the experiment.

### Viability assessment via cell counting in PEG10 overexpressing SKNMC SAM cells

The cells were seeded in 12 well plates (Sarstedt) and transduced with ca. 70% transduction efficiency. The cells with inducible PEG10 expression were also seeded in 12 well plates and incubated with varying concentrations of doxycycline that was replaced every 48 h. The viability of cells was then evaluated by counting trypan blue (0.4%, ThermoFischer Scientific) stained cells with TC20 Automated Cell Counter (BioRad). The counting was done with quadruple technical replicates.

### Microscopy

The cells were seeded in six well plates and Olympus CKX41 microscope equipped with the Philips C550W-4 camera was used to obtain the images. The images were analyzed in ImageJ.

### Cell lysis and western blotting

Cells were lysed in a modified RIPA buffer (50 mM Tris–Cl (pH 7.5), 150 mM NaCl, 1% NP-40, 0.5% Na-deoxycholate, 1 mM EGTA, 0.1% SDS, 50 mM NaF, 10 mM sodium β-glycerolphosphate, 5 mM sodium pyrophosphate, and 1 mM sodium orthovanadate and supplemented with Complete Mini Protease Inhibitor cocktail (Merck)), flash-frozen in liquid nitrogen and cleared via centrifugation. The samples were run for separation on 4–12% BisTris NuPAGE pre-cast gels (ThermoFisher Scientific) and transferred to nitrocellulose membranes by Trans-Blot Turbo Transfer System (BioRad). The membranes were then blocked for 1 h in 5% milk or BSA in TBS-T (100 mM NaCl, 10 mM Tris, pH 7.6, 0.1% Tween 20) and cut prior to incubation with primary antibodies at appropriate dilution overnight at 4 °C. After washing with TBS-T the membranes were incubated in corresponding horseradish peroxidase (HRP)-linked secondary antibody at room temperature for 1 h. Proteins on the membranes were detected via chemoluminescence induced by ECL detection reagent (GE Healthcare) or SuperSignal Western blotting reagent (ThermoFisher Scientific) using the ChemiDoc Imaging system (Biorad). The blots were quantified using Image Lab version 6.1 software (BioRad) and the data were analyzed using Prism GraphPad version 8. Full-length blots of blots used in figures are presented in Supplementary Fig. S7.

## Supplementary Information

Supplementary Information 1.

Supplementary Information 2.

Supplementary Table S1.

Supplementary Table S2.

Supplementary Table S3.

Supplementary Table S5.

## Data Availability

Analysis of publically available EWS-FLI1 knockout studies is summarized in Supplementary Table S1A. Analysis of the NGS data obtained in this study is summarized in Supplementary Tables S1B,C and S2. PEG10 mRNA expression levels across 15 EWS-FLI1 positive Ewing sarcoma cell lines with EWS-FLI knockdown were extracted from published data set in the Ewing Sarcoma Cell Line Atlas. The raw NGS sequencing data for the LIBerty screen in SKNMCs can be found at the Sequence Read Archive (NIH), BioProject accession number PRJNA796551.
