# Author Correction: CRISPR activation screen identifies TGFβ-associated PEG10 as a crucial tumor suppressor in Ewing sarcoma

**DOI:** 10.1038/s41598-022-17582-5

**Published:** 2022-08-02

**Authors:** Vadim Saratov, Quy A. Ngo, Gloria Pedot, Semjon Sidorov, Marco Wachtel, Felix K. Niggli, Beat W. Schäfer

**Affiliations:** 1 Department of Oncology and Children’s Research Center, University Children’s Hospital, Steinwiesstrasse 32, 8032 Zurich, Switzerland; 2 Experimental Infectious Diseases and Cancer Research, Children’s Research Center, University Children’s Hospital of Zurich, University of Zurich, Zurich, Switzerland

Correction to: *Scientific Reports* 10.1038/s41598-022-12659-7, published online 23 June 2022

The original version of this Article contained an error in the Author Contributions section.

“V.S., B.W.S. and F.K.N. designed the study. V.S. designed, performed and analyzed the experiments. Q.A.N. performed bioinformatic analysis of the data from EWS-FLI1 knockout studies, and provided support during NGS data analysis via PinAPL-Py. G.P. and S.S. helped with FACS for the establishment of cell lines. M.W. provided support with experimental design. V.S. and B.W.S. wrote the original manuscript. V.S., B.W.S., S.S., G.P., Q.A.N. and M.W. revised the manuscript. All authors reviewed the results, revised the manuscript and approved the final version of the manuscript.”

now reads:

“V.S., B.W.S. and F.K.N. designed the study. V.S. designed, performed and analyzed the experiments. Q.A.N. performed bioinformatic analysis of the data from EWS-FLI1 knockout studies and provided support during NGS data analysis via PinAPL-Py. G.P. and S.S. helped with FACS for the establishment of cell lines. M.W. provided support with experimental design. V.S and B.W.S. wrote the original manuscript. V.S, B.W.S., S.S., G.P., Q.A.N and M.W. revised the manuscript. All authors reviewed the results and approved the final version of the manuscript.”

The original Article has been corrected.

