# Supplementary material for: CRISPR activation screen identifies TGFβ-associated PEG10 as a crucial tumor suppressor in Ewing sarcoma
Source: Sci Rep. 2022 Jun 23;12:10671. doi: 10.1038/s41598-022-12659-7 (PMC9225990; doi:10.1038/s41598-022-12659-7)
Supplement: Supplementary file 2 — Supplementary Information 2. [file 41598_2022_12659_MOESM2_ESM.pdf]

Supplementary information

CRISPR activation screen identifies TGFβ-associated PEG10 as a crucial tumor suppressor in Ewing sarcoma

Vadim Saratov[1], Quy A. Ngo[1], Gloria Pedot[1], Semjon Sidorov[2], Marco Wachtel[1], Felix K. Niggli[1], *Beat W. Schäfer[1]

[1]Department of Oncology and Children’s Research Center, University Children’s Hospital, Steinwiesstrasse 32, 8032 Zurich, Switzerland.
[2]Experimental Infectious Diseases and Cancer Research, Children's Research Center, University Children's Hospital of Zurich, University of Zurich, Zurich, Switzerland.

***Corresponding author:** Beat W. Schäfer, Department of Oncology, Children’s Hospital Zurich, Steinwiesstrasse 75 8032 Zurich, Switzerland, Email: beat.schaefer@kispi.uzh.ch, Phone: +41 44 266 75 53

Supplementary Figures and Figure Legends S1-S7

The Supplementary Figures support data from the following main figures: S1 to Fig. 1, S2 and S3 to Fig. 2, S4 to Fig. 4, S5 to Fig. 5, S6 to Fig. 6. Supplementary figure legends are included below each supplementary figure.

Full-length blots of main and supplementary western blot images are presented in Supplementary Fig. S7

Supplementary Data S1 - 2

The Supplementary Data 1 contains settings used in PinAPL-Py to analyze the NGS data.

The Supplementary Data 2 contains raw read count data calculated via PinAPL-Py as part of the analysis of the NGS data.

Supplementary Tables S1-S5

The Supplementary Table S1 contains LIBerty gene list and rankings. The Supplementary Table S2 contains LIBerty gRNA list and rankings. The Supplementary Table S3 contains gRNA 1 sequences from the LIBerty library. The Supplementary Table S4 contains list of antibodies used in this study. The Supplementary Table S5 contains list of primers used in this study. Tables S1-S3 and S5 as separate Excel files.

Supplementary Figures

A

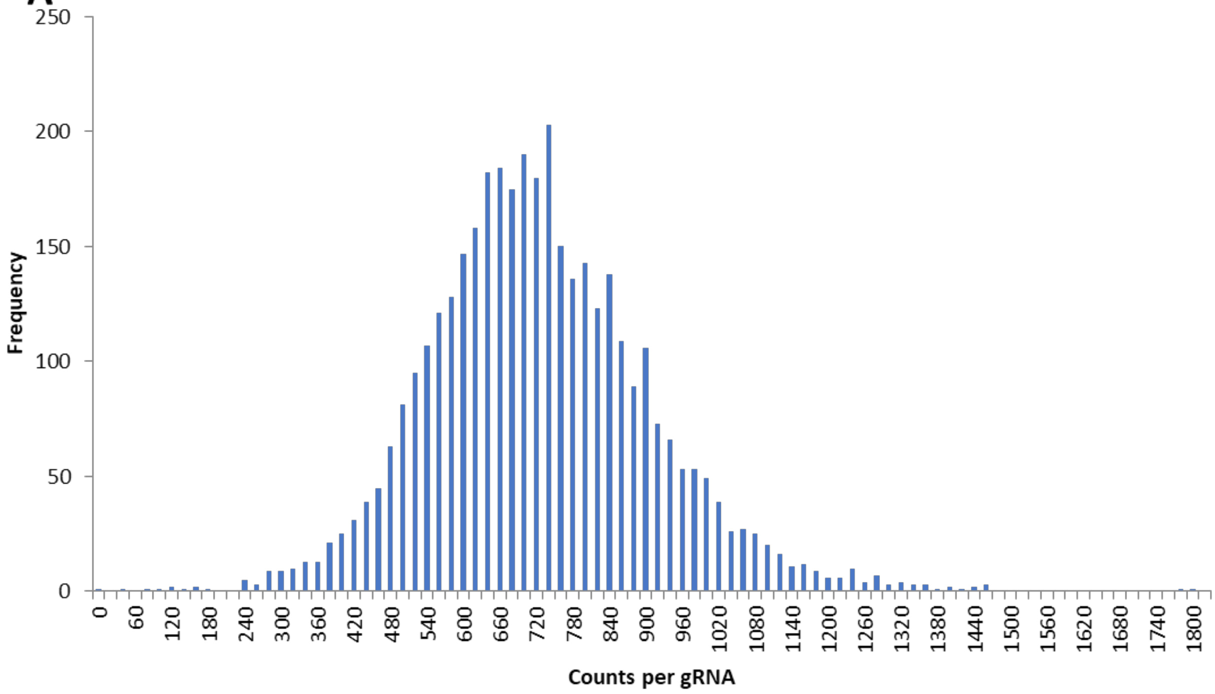

B

Aim according to guidelines	LIBerty library
>70% perfectly matching	3776 (100.0%)
<0.5% undetected	1 (0.03%) missing
<10 skew ratio<br>(top 10%/bottom 10%)	2.6

Supplementary Figure S1. Quality check of the LIBerty library in plasmid format.

A, NGS analysis of the plasmid LIBerty library for frequency of counts per gRNA. **B**, Comparison of the suggested guidelines for custom libraries to LIBerty library data.

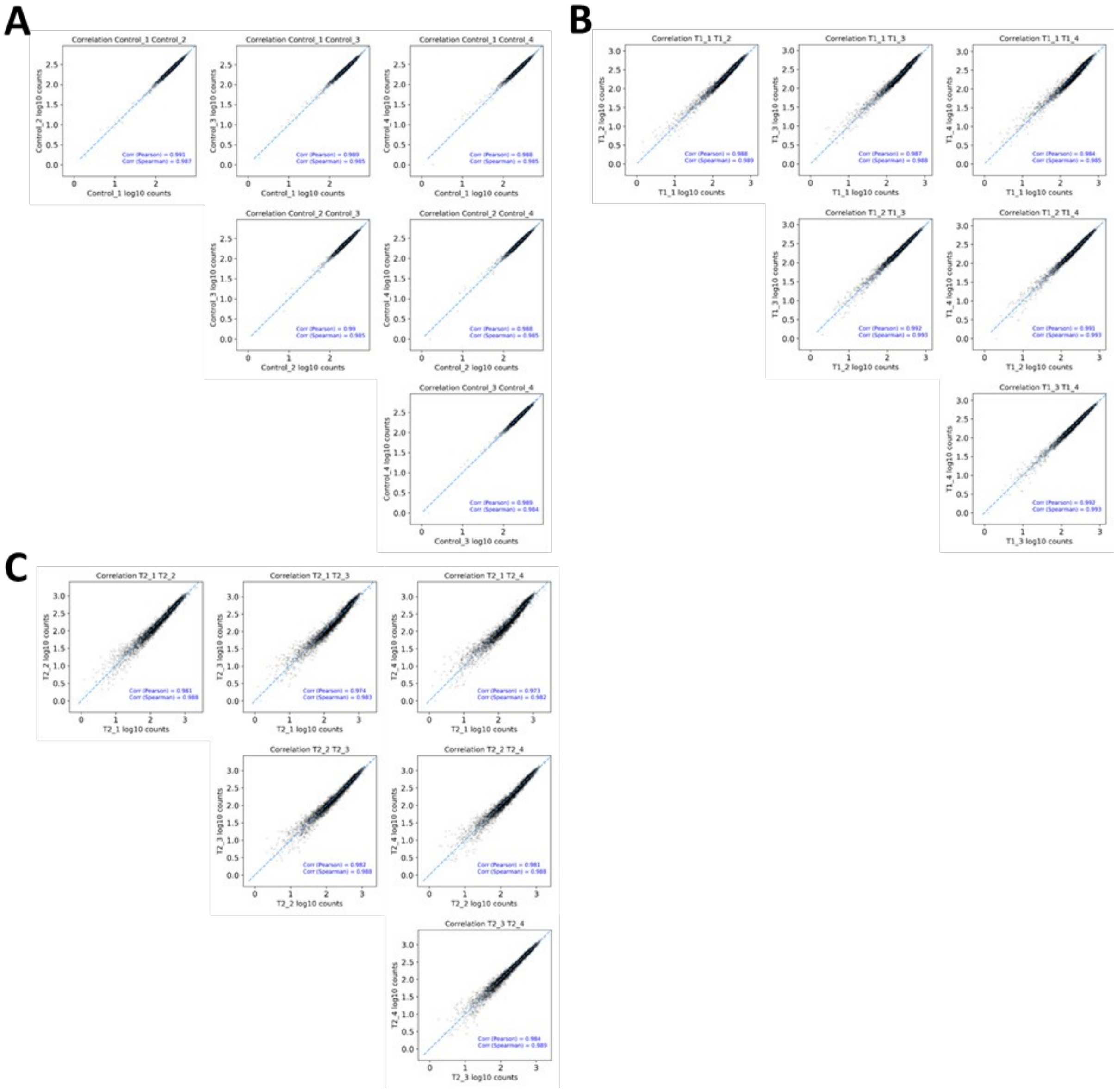

Supplementary Figure S2. Correlation of screen replicates.

A, Correlation of NGS data points within samples collected on day three post transduction used for further analysis as control or time point zero. **B**, Correlation of NGS data points within samples collected on day ten post transduction used for further analysis as time point one. **C**, Correlation of NGS data points within samples collected on day twenty post transduction used for further analysis as time point two. All coefficients were calculated by PinAPL-Py pipeline. Image was created by using PinAPL-Py version 2.8.1, pinapl-py.ucsd.edu.

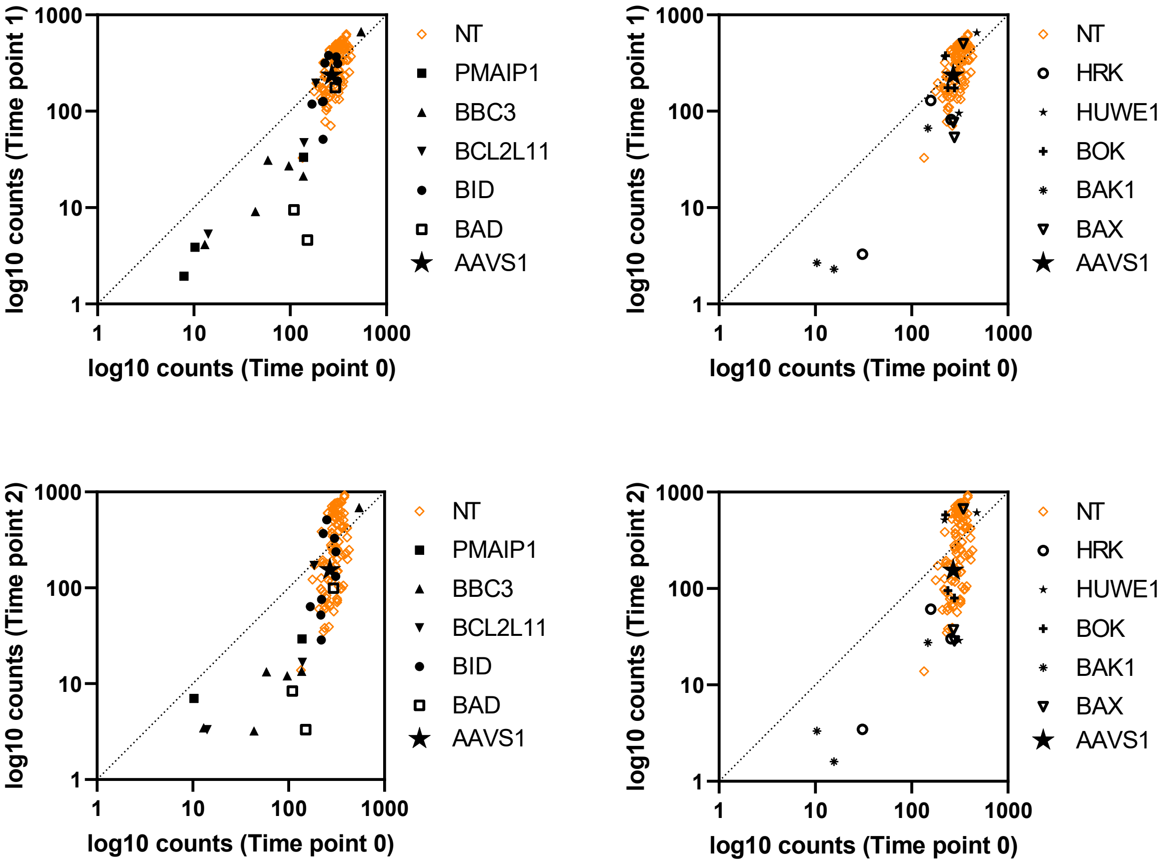

Supplementary Figure S3. gRNA counts of control gRNAs compared to time point zero.

NGS analysis of the CRISPRa screen was done via PinAPL-Py with gRNA counts as main readout. **A-B**, gRNA counts of each unique gRNA from time point one compared to time point zero with non-targeting gRNAs in orange, AAVS1 and positive controls labeled according to the legend. **C-D**, gRNA counts of each unique gRNA from time point two compared to time point zero with non-targeting gRNAs in orange, AAVS1 and positive controls labeled according to the legend. All subfigures were created by using GraphPad version 8, www.graphpad.com.

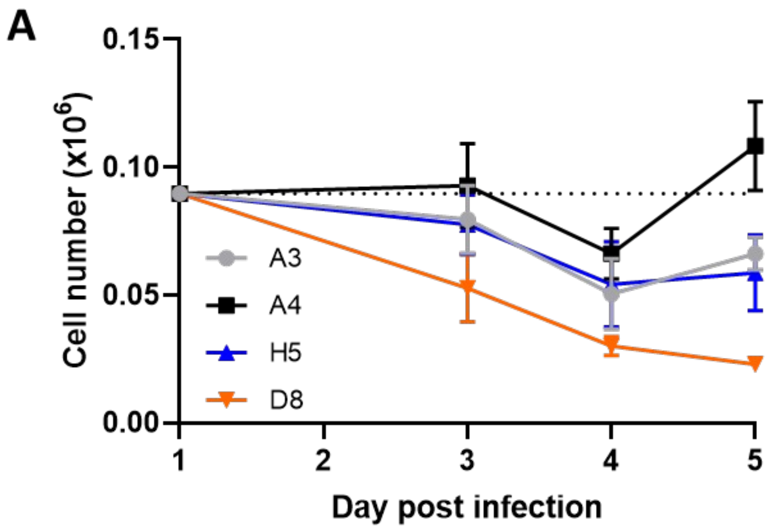

Supplementary Figure S4. Cell numbers decline in SKNCM SAM clonal cell lines overexpressing PEG10.

A, Quantification of the number of PEG10 overexpressing SKNMC SAM cells with the seeding amount highlighted via the dotted line. Image was created by using GraphPad version 8, www.graphpad.com.

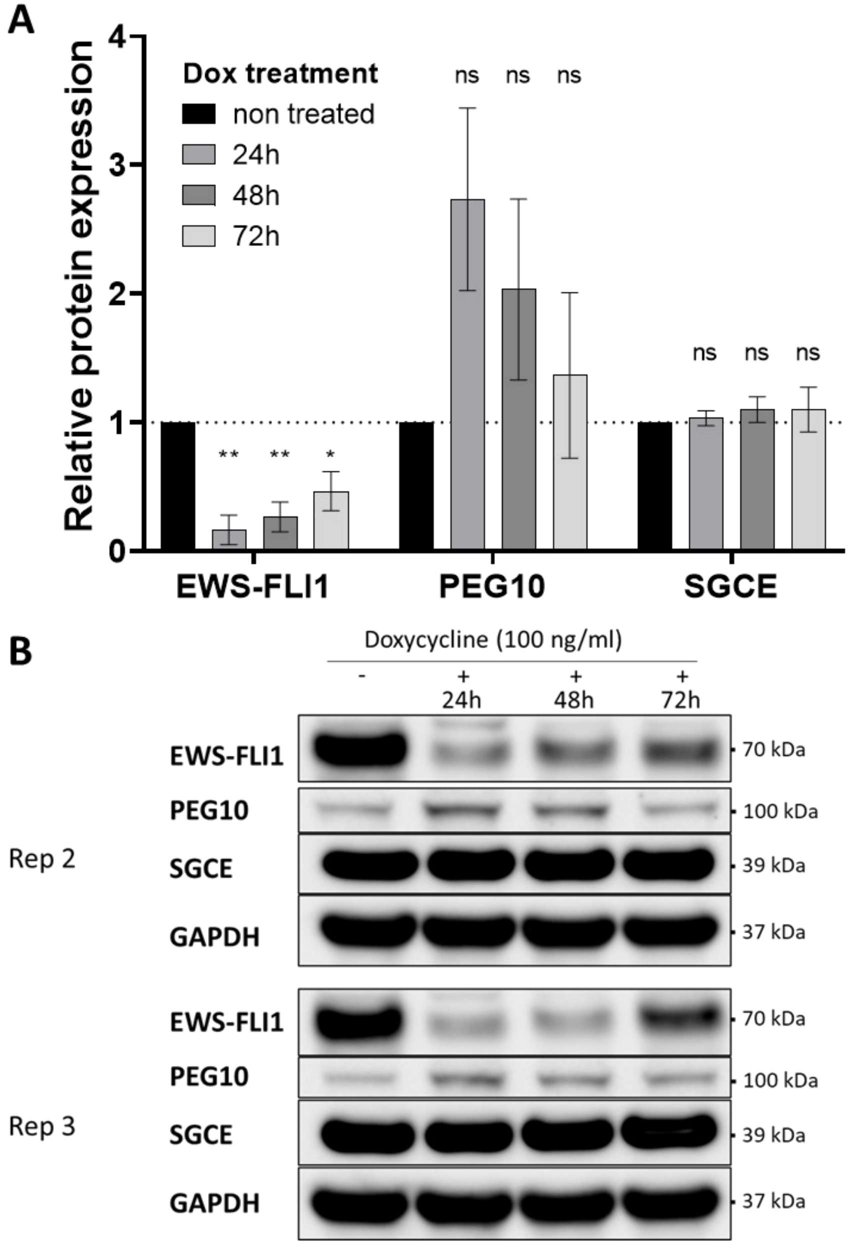

Supplementary Figure S5. Densitometry analysis and replicates of EWS-FLI1 knock down in SKNMCs.

A, Densitometry of the Western Blot measuring EWS-FLI1, PEG10 and SGCE protein levels in SKNMC cells with the inducible EWS-FLI1 shRNA construct upon induction for 24, 48 and 72 hours (n=3, * $p< 0.1$, ** $p < 0.01$, *** $p < 0.001$, paired t-test). β-tubulin was used to normalize relative protein expression. Image was created by using GraphPad version 8, www.graphpad.com. **B**, Biological replicates of the Western Blot measuring EWS-FLI1, PEG10 and SGCE protein levels in SKNMC cells with the inducible EWS-FLI1 shRNA construct upon induction for 24, 48 and 72 hours. Image was created by using Image Lab version 6.1, www.bio-rad.com/en-ch/product/image-lab-software.

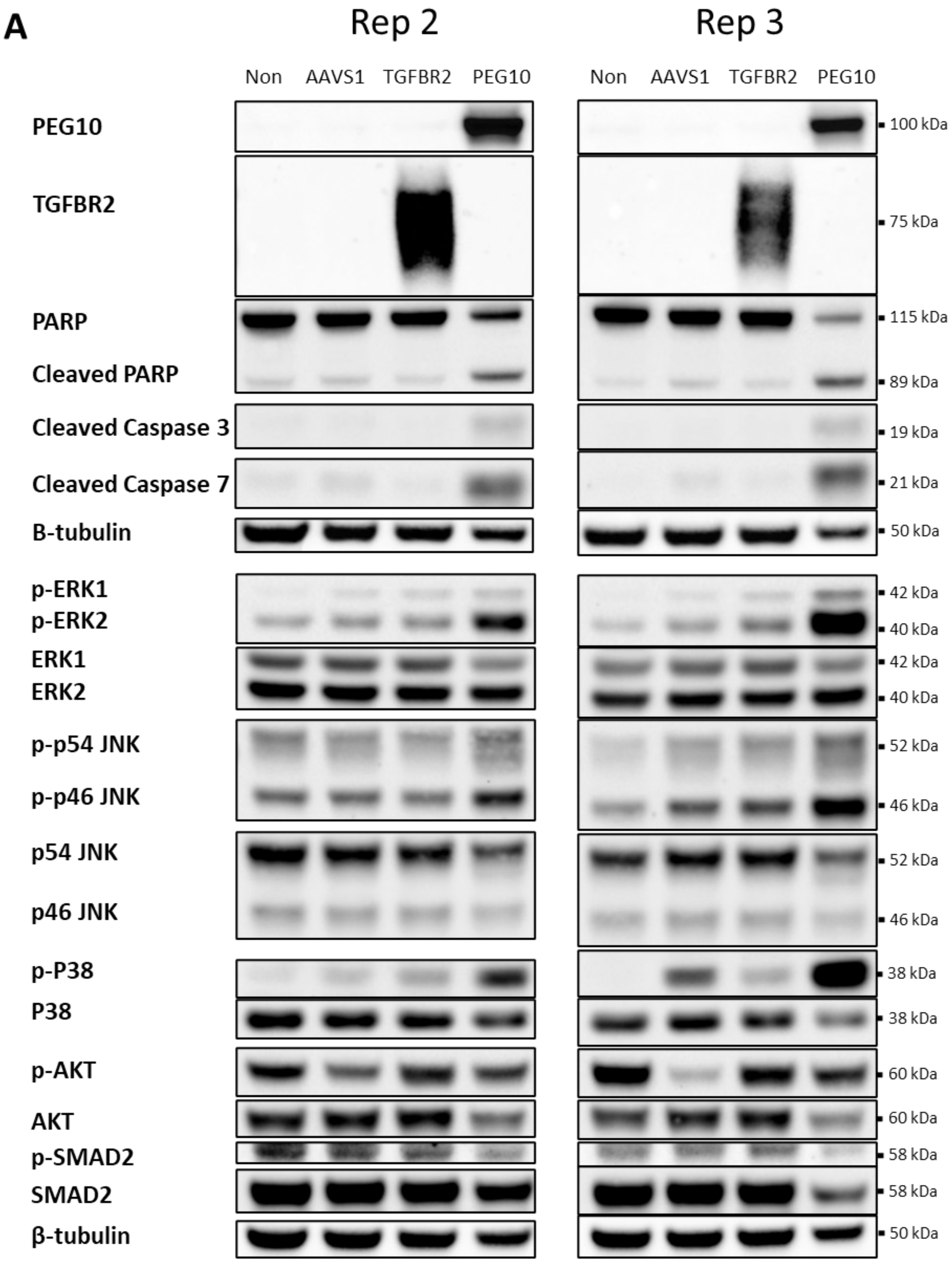
A
Rep 2
Rep 3
Non
AAVS1
TGFBR2
PEG10
Non
AAVS1
TGFBR2
PEG10
PEG10
TGFBR2
PARP
Cleaved PARP
Cleaved Caspase 3
Cleaved Caspase 7
B-tubulin
p-ERK1
p-ERK2
ERK1
ERK2
p-p54 JNK
p-p46 JNK
p54 JNK
p46 JNK
p-P38
P38
p-AKT
AKT
p-SMAD2
SMAD2
β-tubulin
100 kDa
75 kDa
115 kDa
89 kDa
19 kDa
21 kDa
50 kDa
42 kDa
40 kDa
42 kDa
40 kDa
52 kDa
46 kDa
52 kDa
46 kDa
38 kDa
38 kDa
60 kDa
60 kDa
58 kDa
58 kDa
50 kDa

B

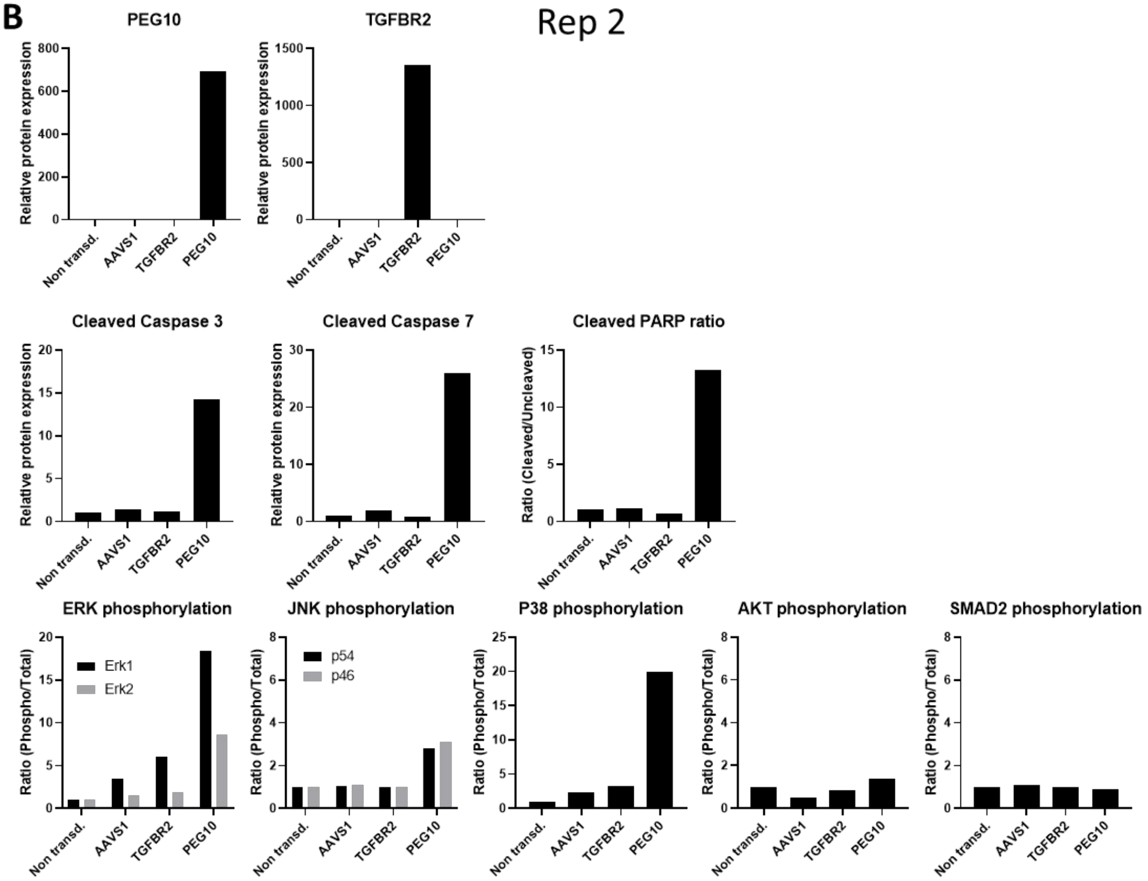
Rep 2
PEG10
TGFBR2
Cleaved Caspase 3
Cleaved Caspase 7
Cleaved PARP ratio
ERK phosphorylation
JNK phosphorylation
P38 phosphorylation
AKT phosphorylation
SMAD2 phosphorylation
Relative protein expression
Ratio (Cleaved/Uncleaved)
Ratio (Phospho/Total)
Erk1
Erk2
p54
p46
Non transd.
AAVS1
TGFBR2
PEG10

C

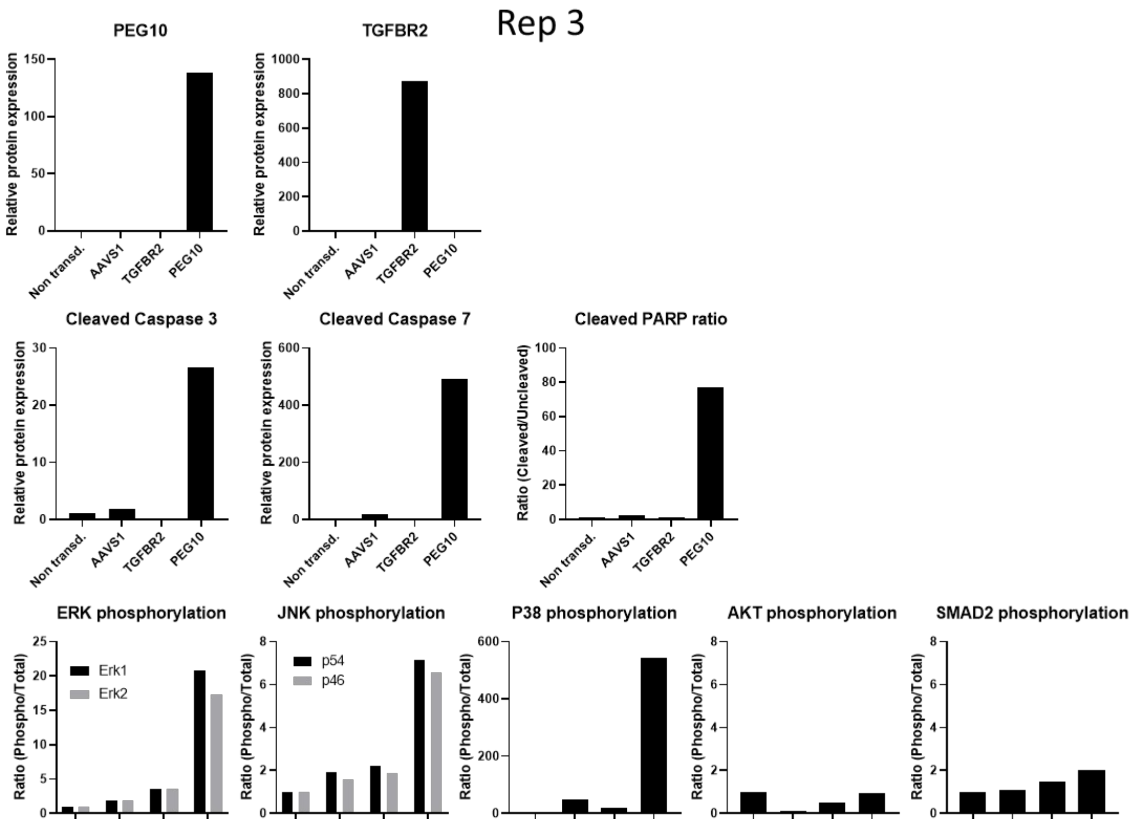
Rep 3
PEG10
TGFBR2
Cleaved Caspase 3
Cleaved Caspase 7
Cleaved PARP ratio
ERK phosphorylation
JNK phosphorylation
P38 phosphorylation
AKT phosphorylation
SMAD2 phosphorylation
Relative protein expression
Ratio (Cleaved/Uncleaved)
Ratio (Phospho/Total)
Erk1
Erk2
p54
p46
Non transd.
AAVS1
TGFBR2
PEG10

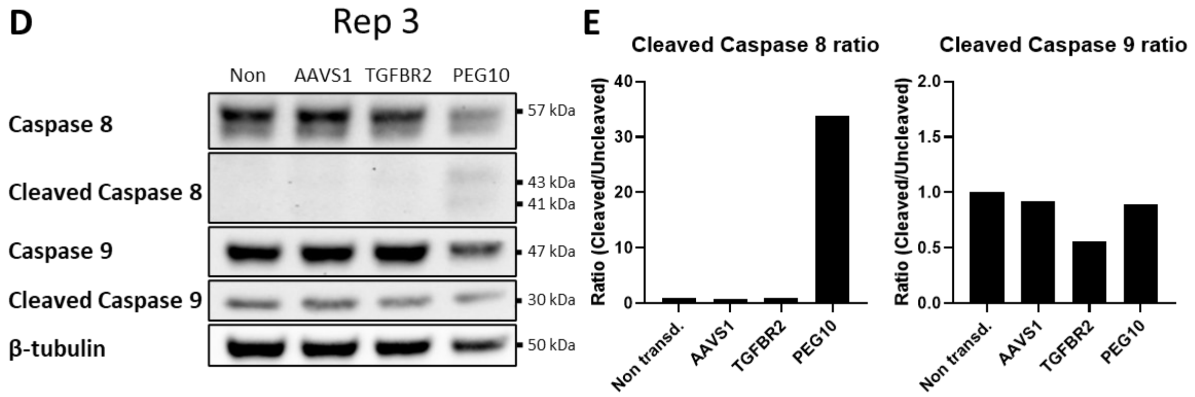

Supplementary Figure S6. Replicates of the signaling investigation of the TGFβ pathways and apoptotic pathway in TGFBR2 and PEG10 overexpressing SKNMC SAM cells.

A, Biological replicates of the Western Blot measuring PEG10, TGFBR2 protein levels, cleavage status of PARP, Caspase 3 and 7, phosphorylation status of ERK, JNK, P38, AKT and SMAD2 in TGFBR2 and PEG10 overexpressing SKNMC SAM cells 4 days post transduction. Image was created by using Image Lab version 6.1, www.bio-rad.com/en-ch/product/image-lab-software.

B, **C**, Densitometry of the Western Blot measuring PEG10, TGFBR2 protein levels, cleavage status of PARP, Caspase 3 and 7, phosphorylation status of ERK, JNK, P38, AKT and SMAD2 in TGFBR2 and PEG10 overexpressing SKNMC SAM cells 4 days post transduction. β-tubulin was used to normalize relative protein expression of PEG10, TGFBR2, Caspase 3 and 7. Cleaved PARP was normalized with total length PARP. Phosphorylated ERK1/2, SAPK/JNK, P38, AKT and SMAD2 were normalized with total ERK1/2, SAPK/JNK, P38, AKT and SMAD2. Image was created by using GraphPad version 8, www.graphpad.com.

D, Biological replicate of the Western Blot measuring cleavage status of Caspase 8 and 9 in TGFBR2 and PEG10 overexpressing SKNMC SAM cells 4 days post transduction. Image was created by using Image Lab version 6.1, www.bio-rad.com/en-ch/product/image-lab-software.

E, Densitometry of the Western Blot measuring cleavage status of Caspase 8 and 9 in TGFBR2 and PEG10 overexpressing SKNMC SAM cells 4 days post transduction. Cleaved Caspase 8 and 9 were normalized with total length Caspase 8 and 9. Image was created by using GraphPad version 8, www.graphpad.com.

Supplementary Figure S7: Original Western blots used in this study.

Most possible uncropped images of Western blots used in main and supplementary figures. Targets for used antibodies are indicated on the left from the bands. Areas of the blot used in Figures are highlighted by orange delineation. All images were created by using Image Lab version 6.1, www.bio-rad.com/en-ch/product/image-lab-software.

Western blots in Figure 4F

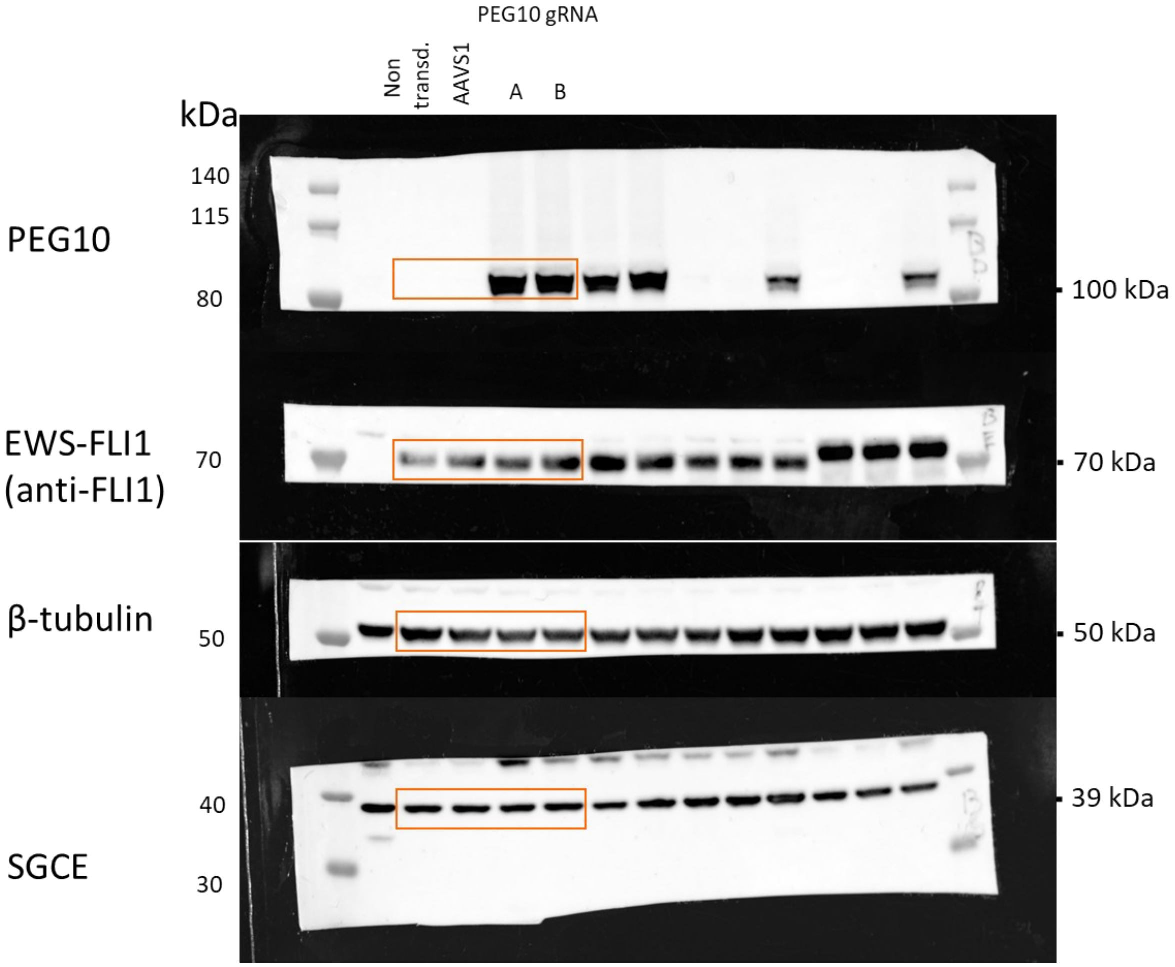

Western blots in Figure 5B

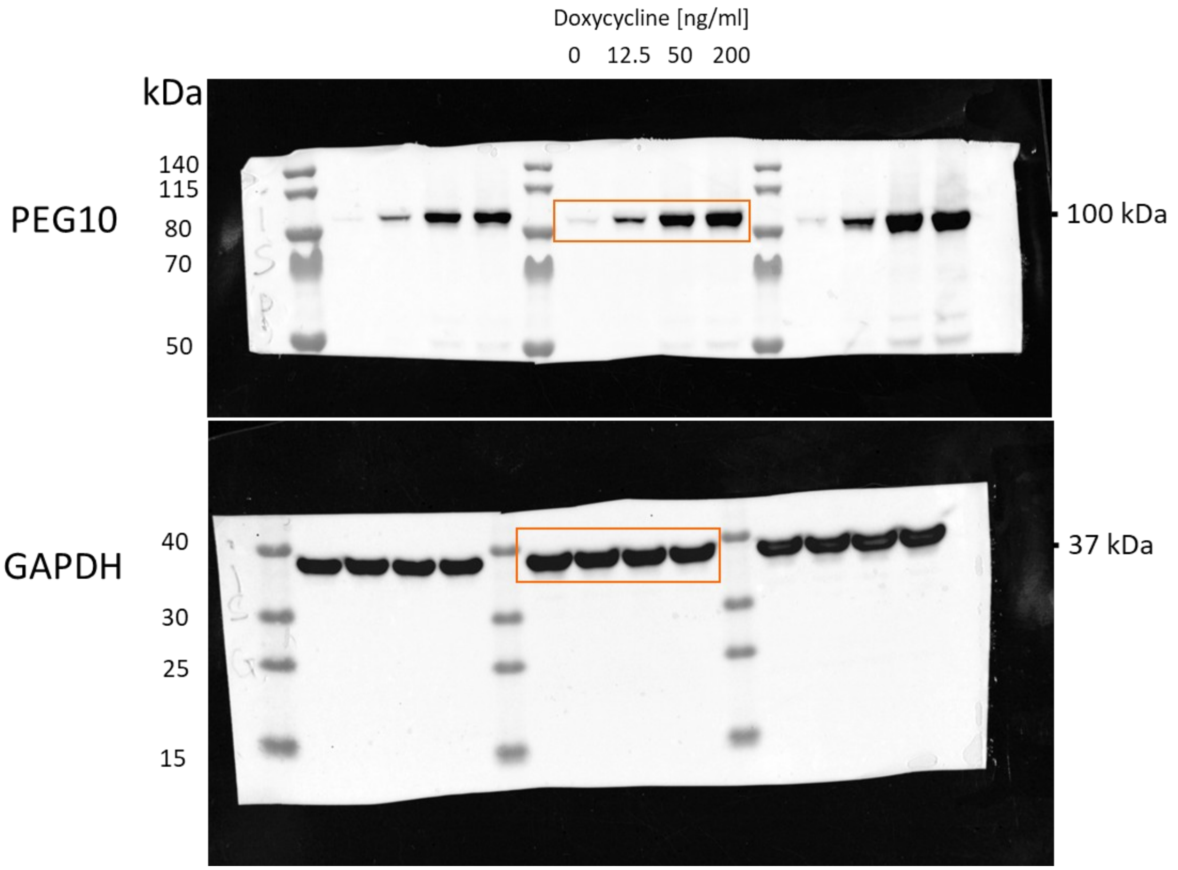

Western blots in Figure 5D

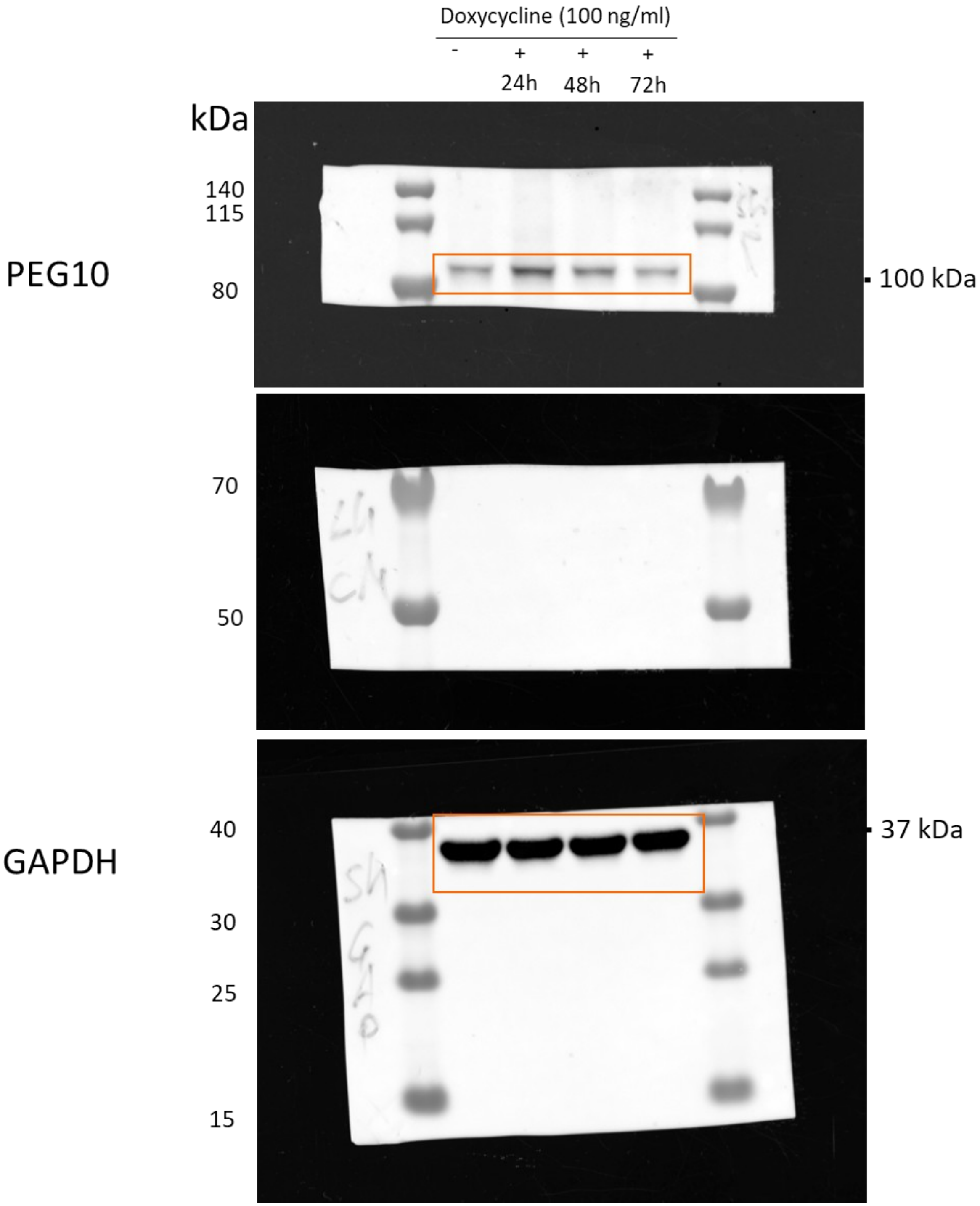

Western blots in Figure 5D

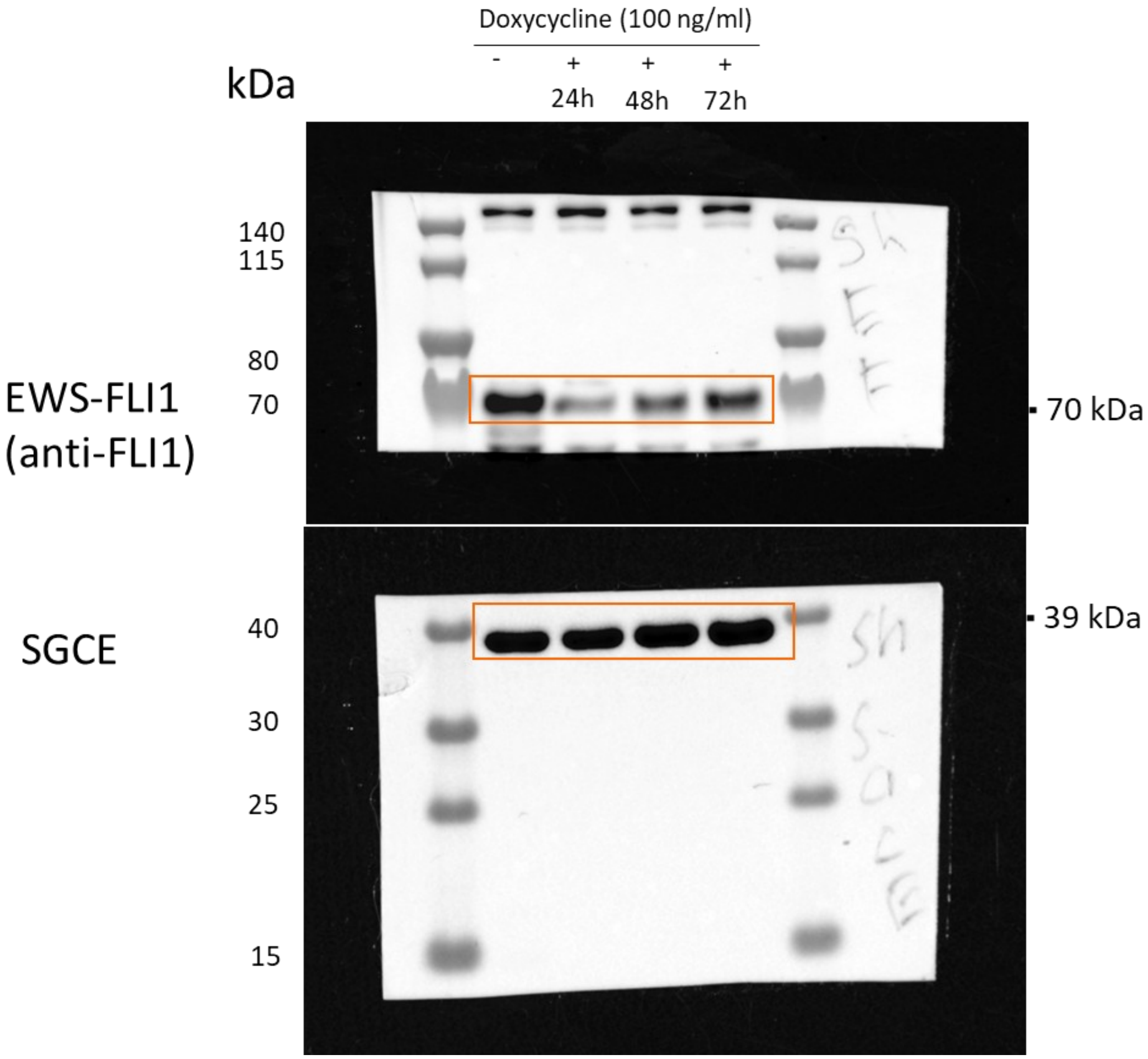

Western blots in Suppl. Figure S4B

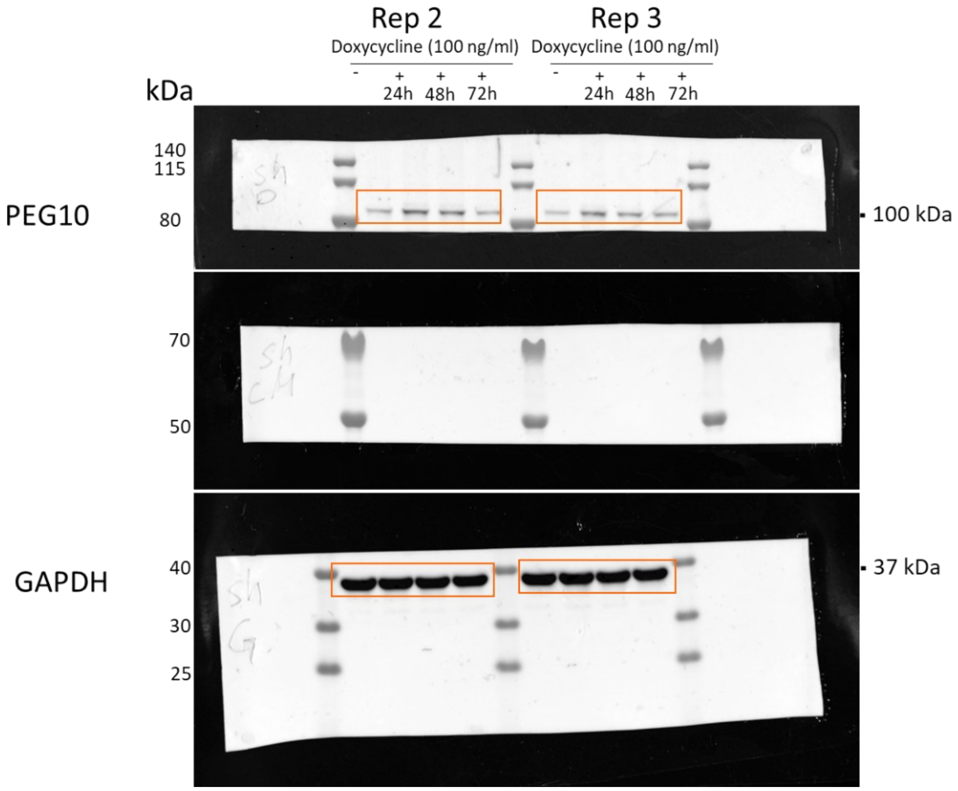

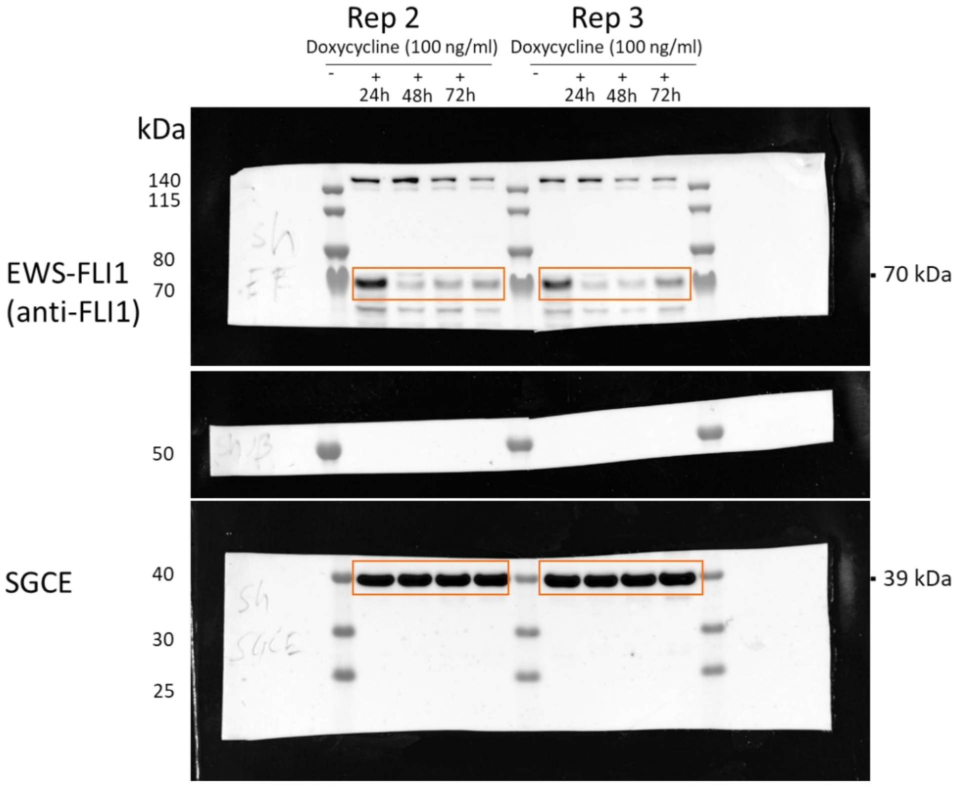

Western blots in Figure 6B

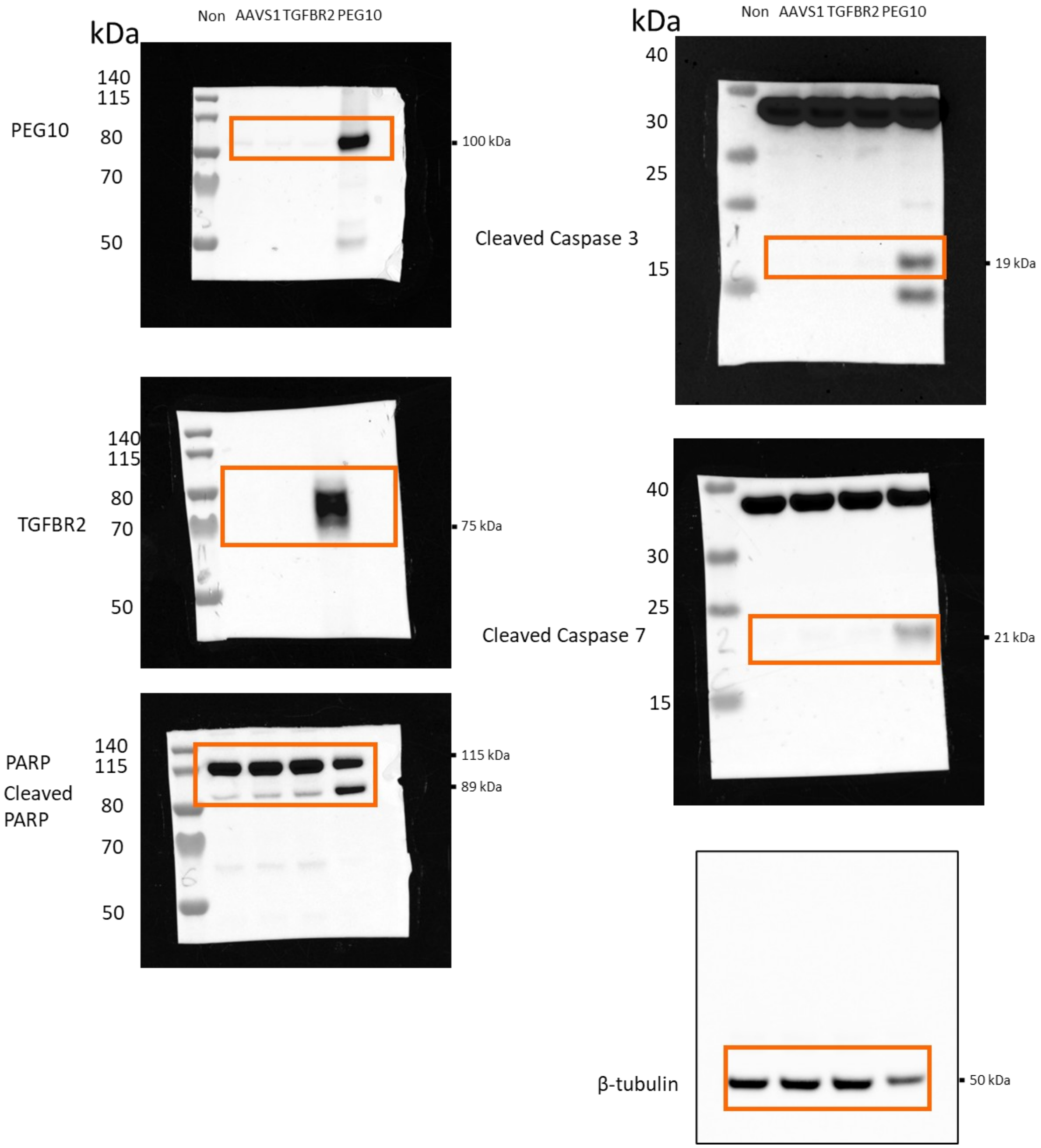

Western blots in Figure 6B

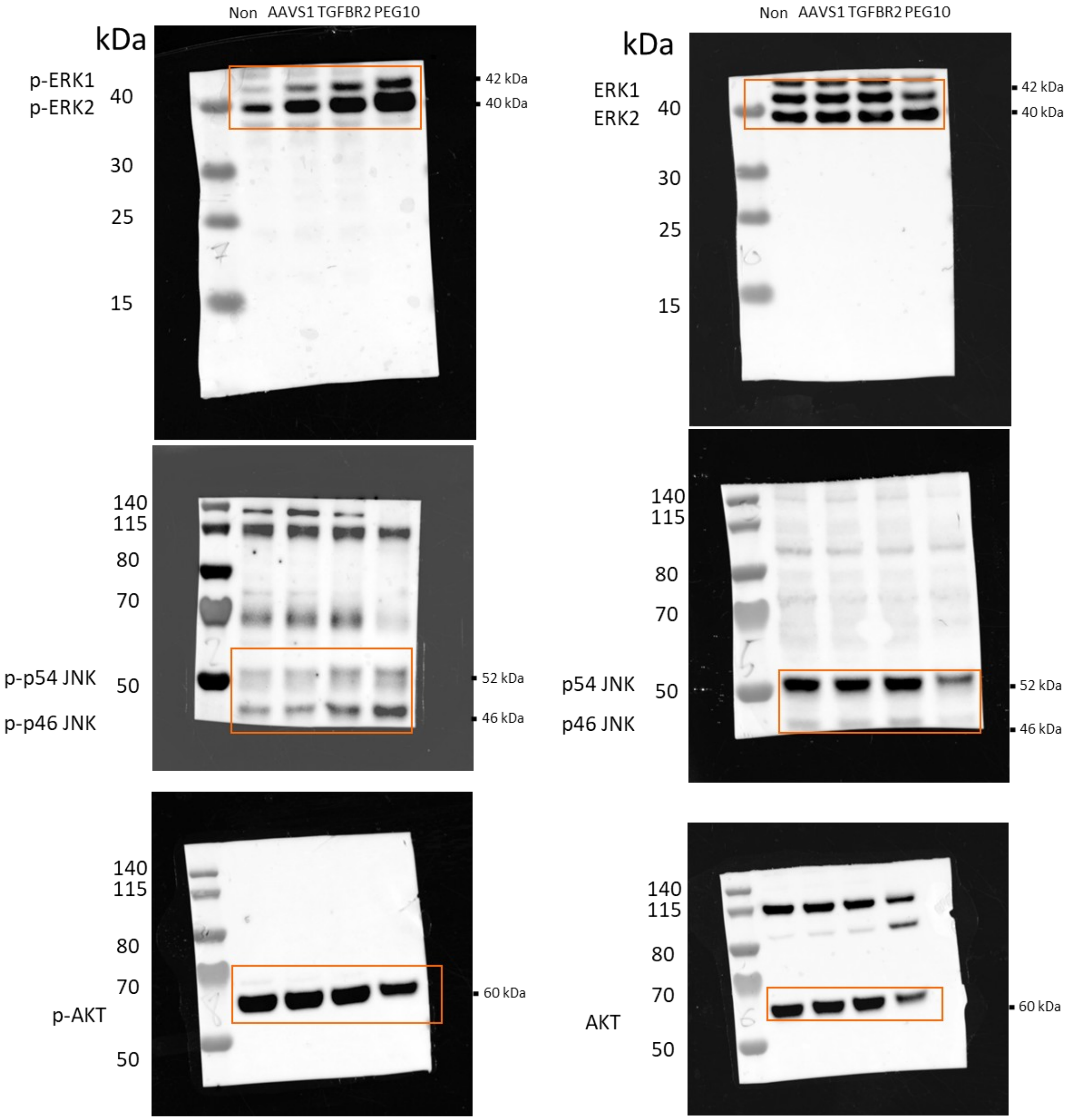

Western blots in Figure 6B

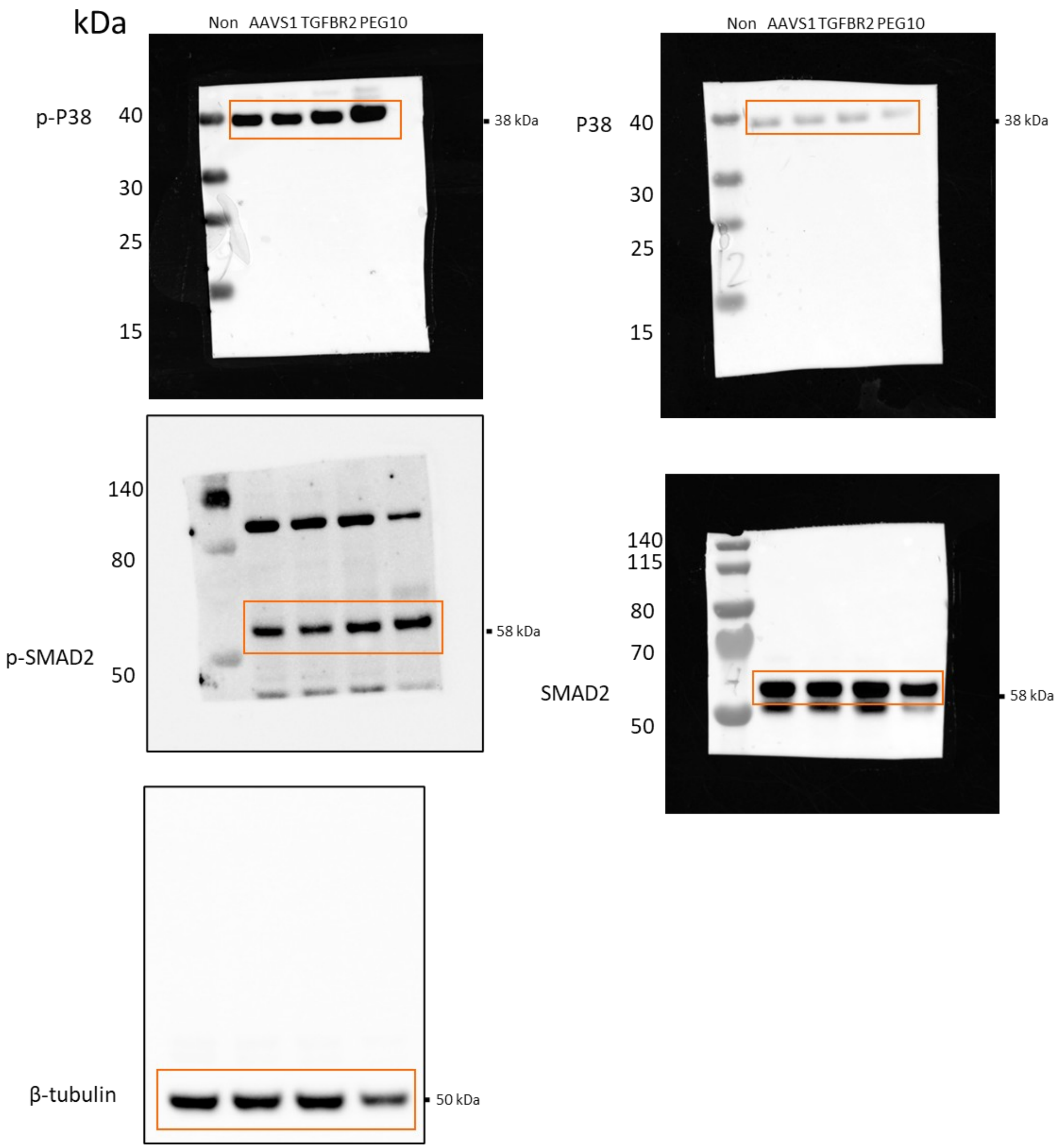

Western blots in Suppl. Figure S5A

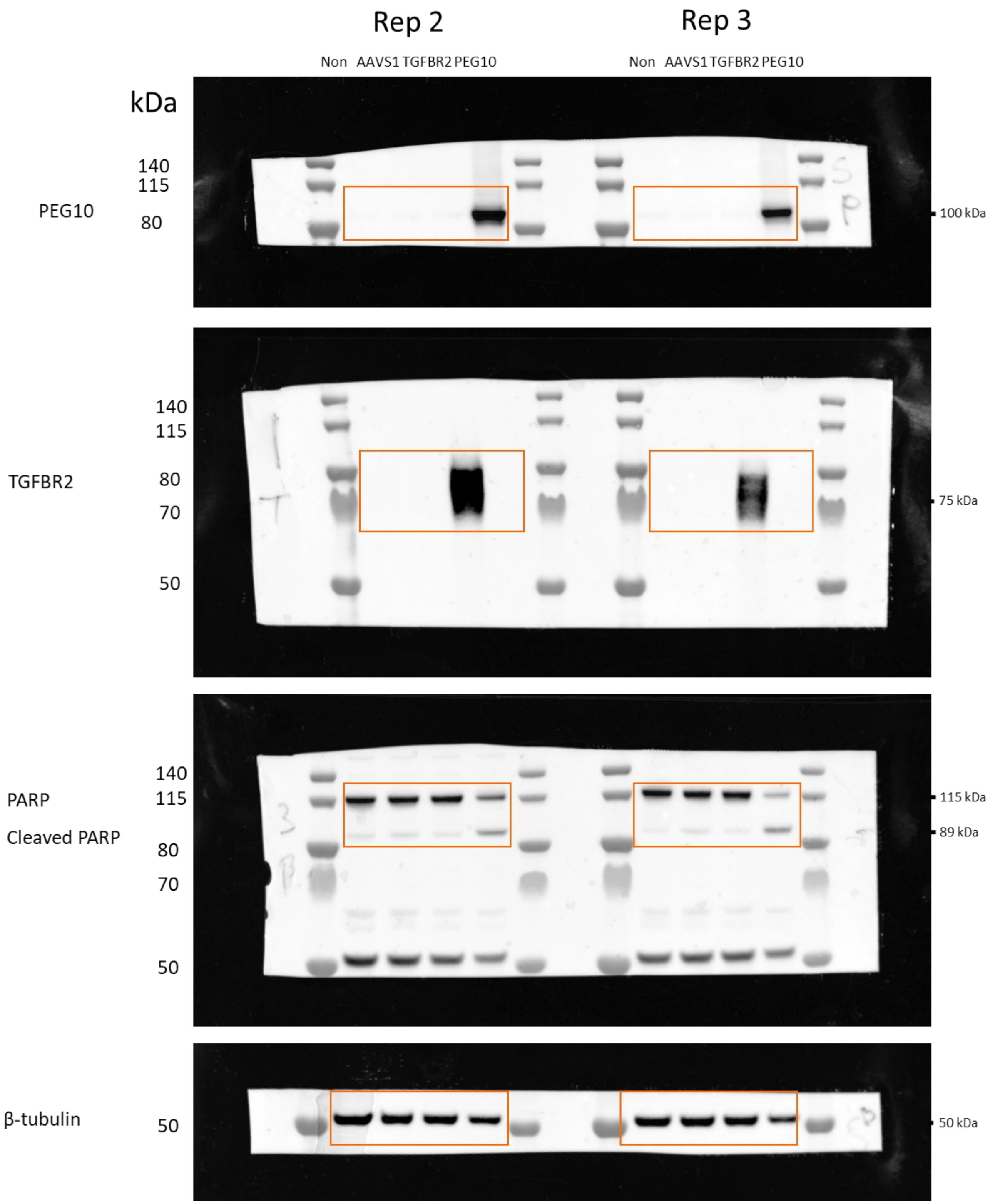

Western blots in Suppl. Figure S5A

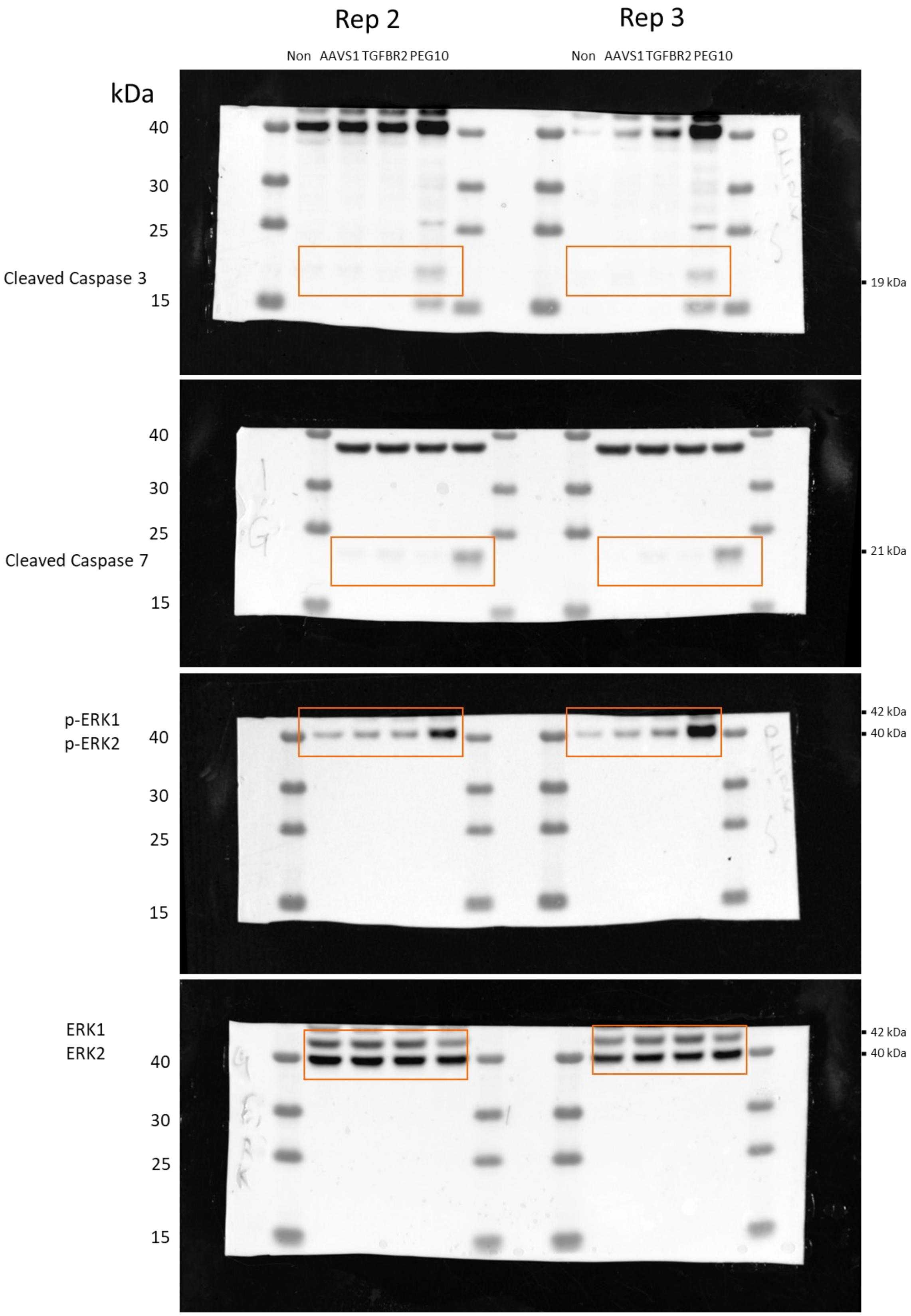

Western blots in Suppl. Figure S5A

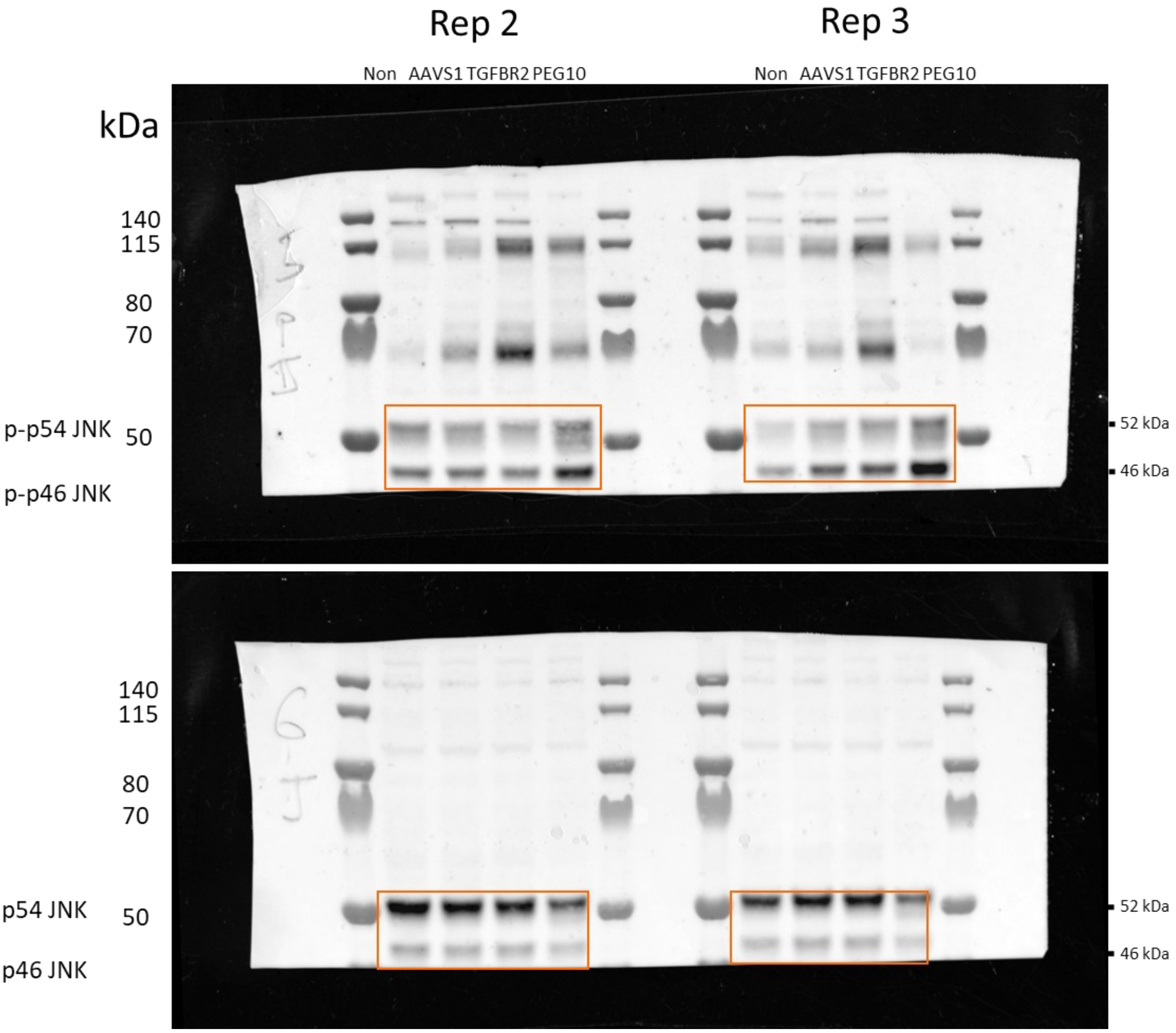

Western blots in Suppl. Figure S5A

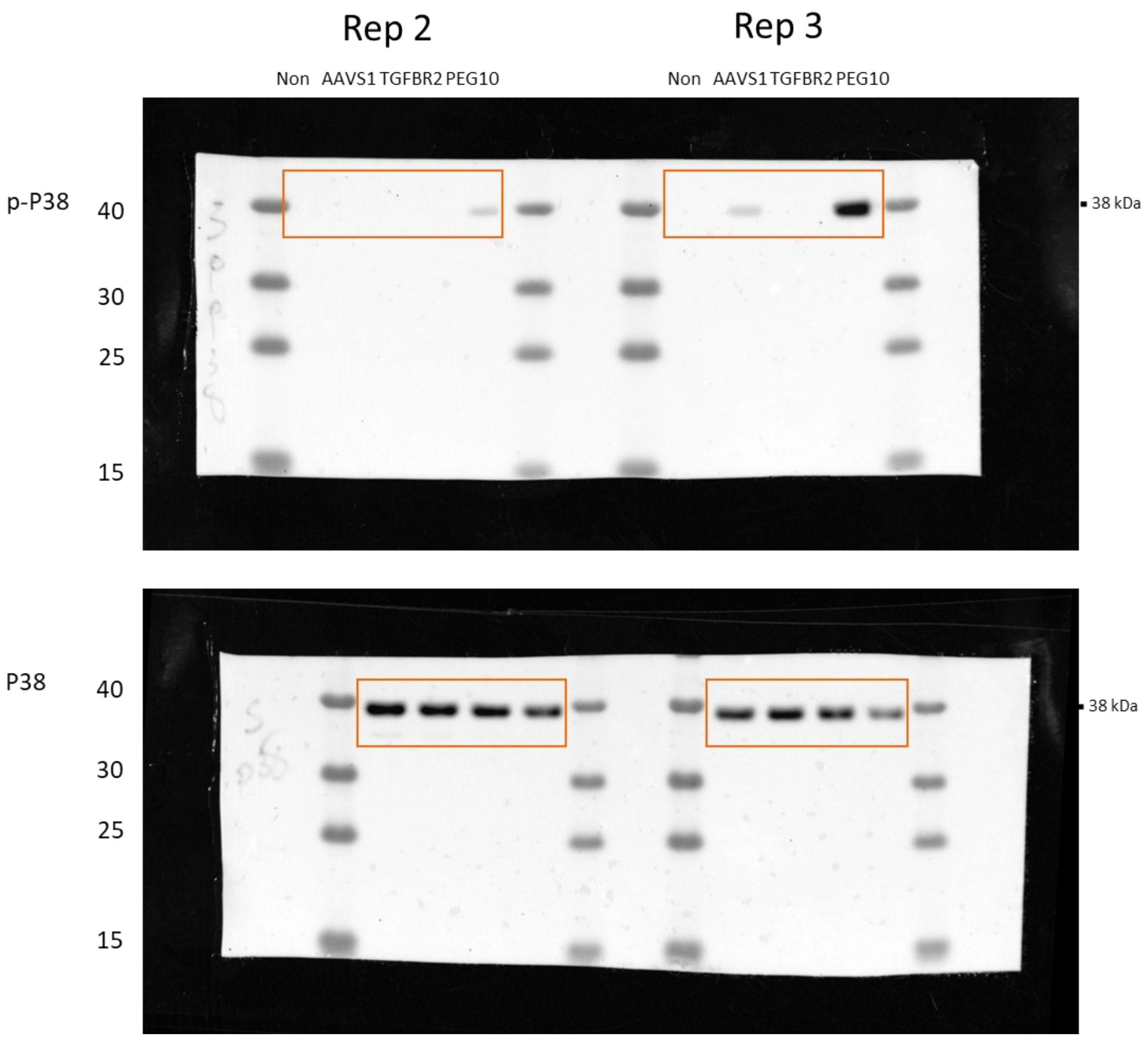

Western blots in Suppl. Figure S5A

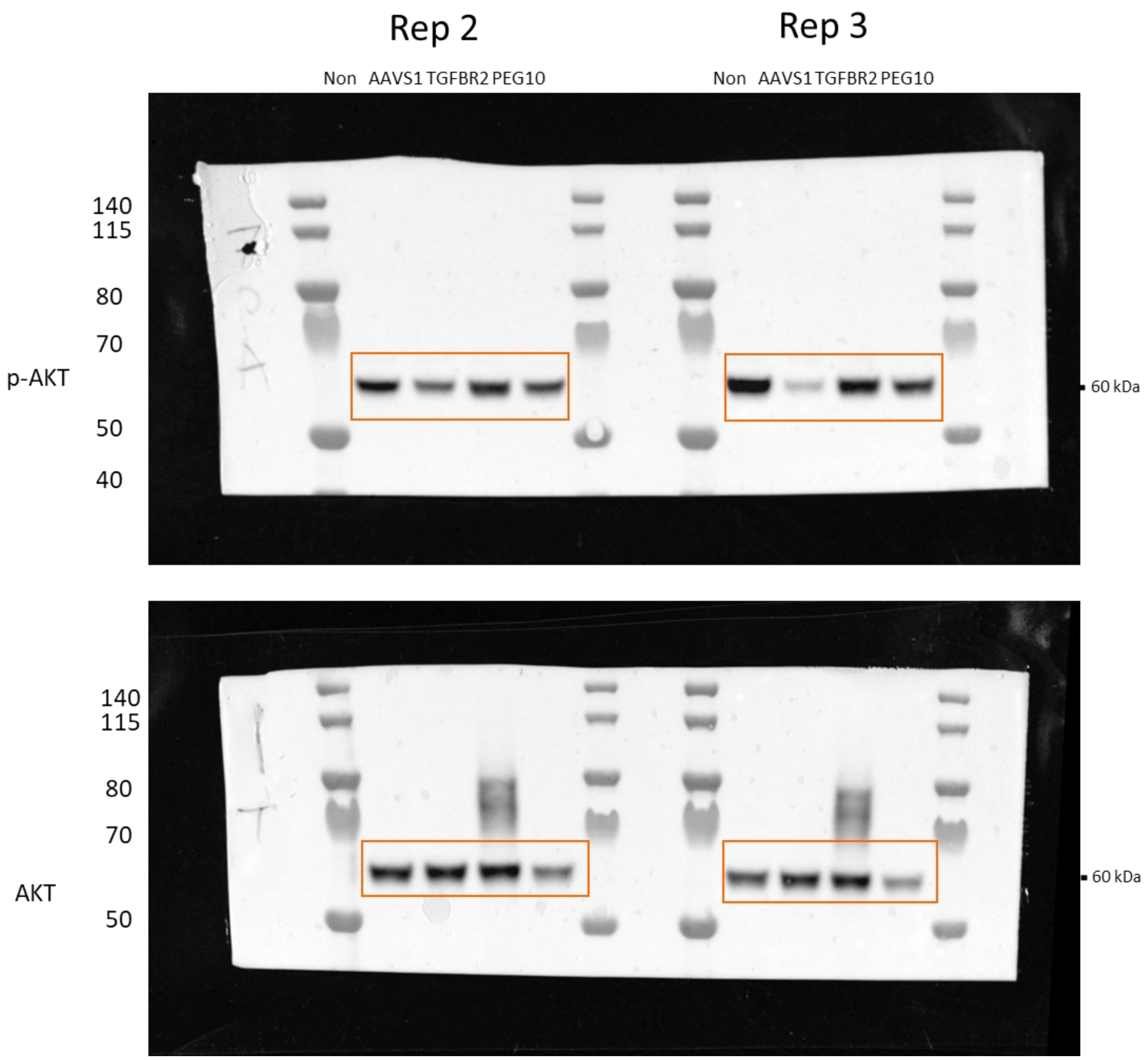

Western blots in Suppl. Figure S5A

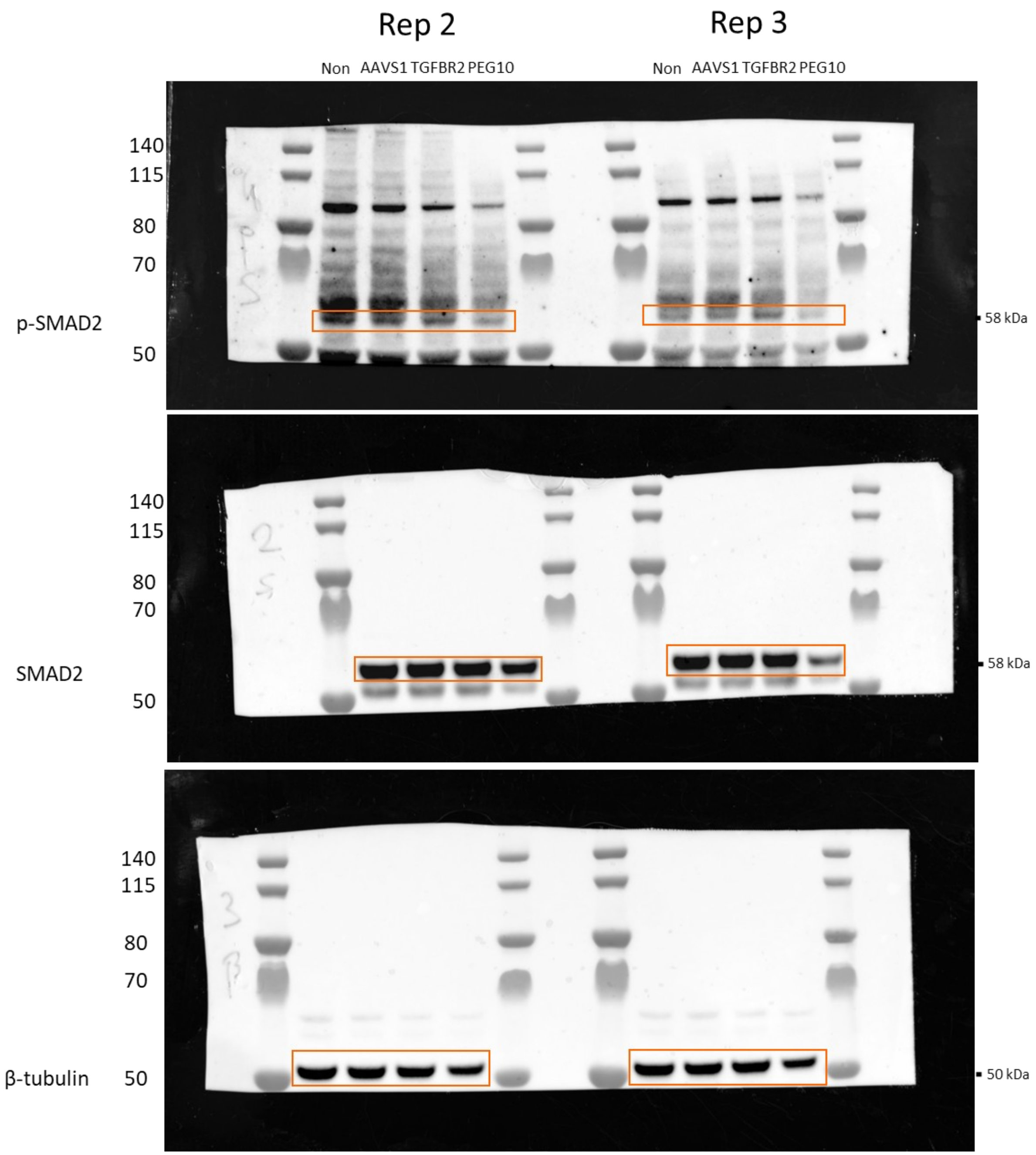

Western blots in Figure 6B and Suppl. Figure S5A

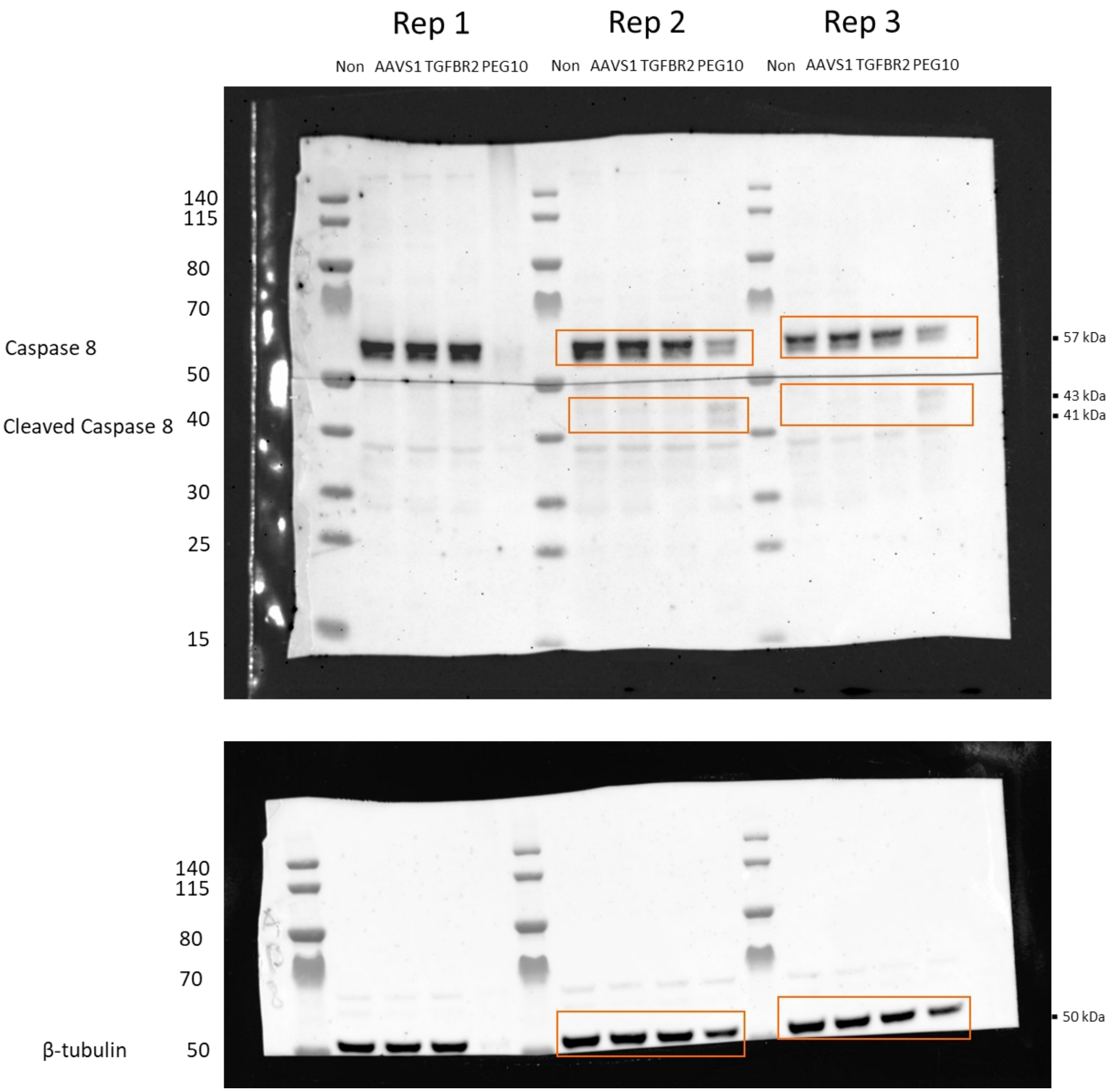

Western blots in Figure 6B and Suppl. Figure S5A

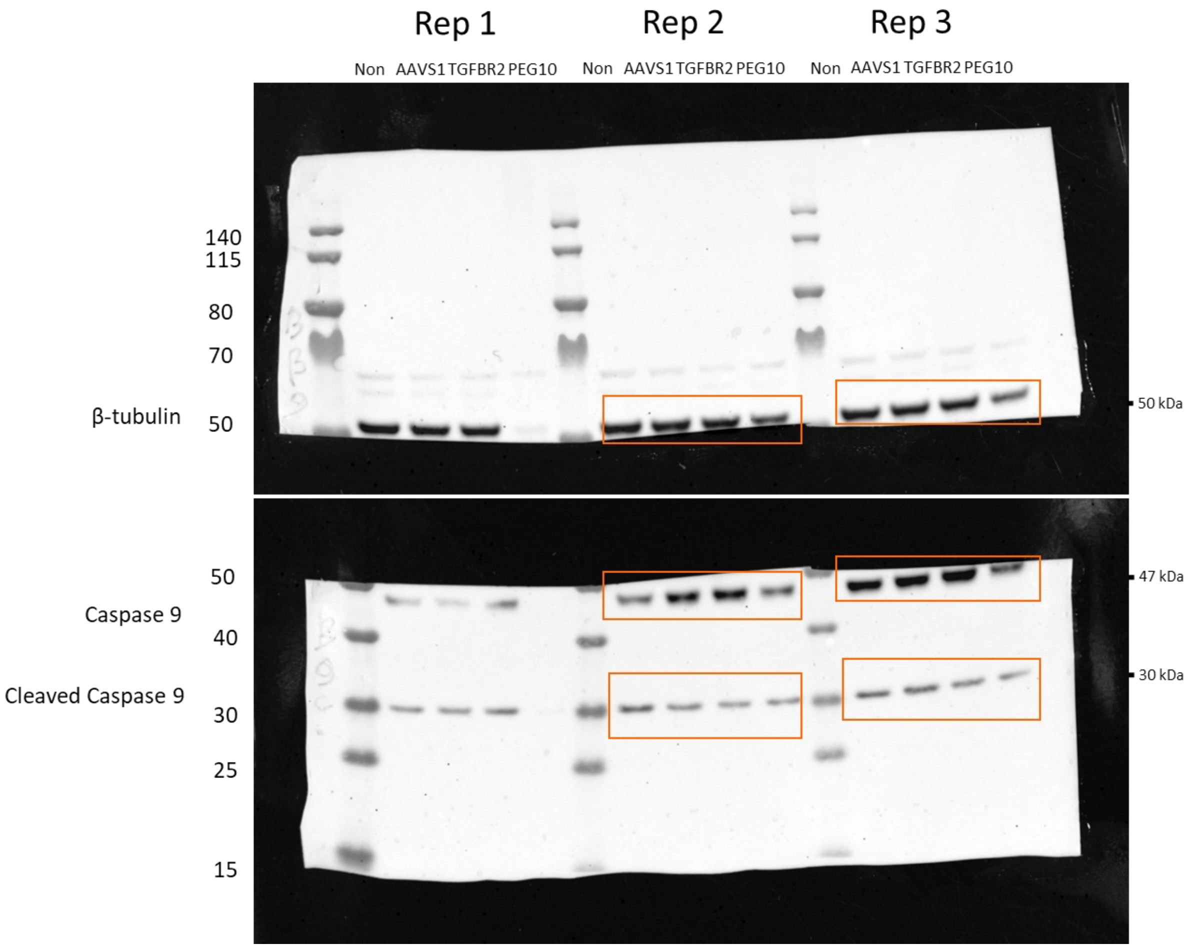

Supplementary Data

Supplementary Data S1: PinAPL-Py settings for the analysis of the NGS data used in this study.

```
# Required
ScreenType: 'depletion'
LibFilename: 'LIBerty_pinapl.tsv'

# Library Parameters
seq_5_end: 'TCTTGTGGAAAGGACGAAACACCN'
NonTargetPrefix: 'Ctrl'
NumGuidesPerGene: 3

# Alignment
sgRNALength: 20
CutErrorTol: 0.1
AS_min: 40
Theta: 2
L_bw: 11
N_bw: 1
i_bw: 'S,1,0.75'
AlnOutput: 'Delete'
keepCutReads: False
delta: 1
R_min: 20

# Read Counting
Normalization: 'cpm'
Cutoff: 0
RoundCount: False
repl_avg: 'median'

# Gene Ranking
GeneMetric: 'aRRA'
Np: 1000
P_0: 0.01
thr_STARS: 10

# Statistical Significance
alpha_s: 0.01
alpha_g: 0.05
padj: 'fdr_bh'
```

```
p_overdisp: 0.01

# Sample Clustering
ClusterBy: 'variance'
TopN: 25

# Visualization
dotsize: 10
TransparencyLevel: 0.1
scatter_annotate: False
ShowNonTargets: False
logbase: 10
width_p: 800
height_p: 800
fontsize_p: 14
marginsize: 10
max_q: 95
svg: True
dpi: 300
HitListFormat: 'tsv'

WorkingDir: '/workingdir/'
DataDir: '/workingdir/Data/'
TempDataDir: '/workingdir/TempData/'
LibDir: '/workingdir/Library/'
IndexDir: '/workingdir/Library/Bowtie2_Index/'
ScriptsDir: '/opt/PinAPL-Py/Scripts/'
AlignDir: '/workingdir/Alignments/'
AnalysisDir: '/workingdir/Analysis/'
TrimLogDir: '/workingdir/Analysis/Read_Trimming'
HitDir: '/workingdir/Analysis/sgRNA_Rankings'
GeneDir: '/workingdir/Analysis/Gene_Rankings'
ControlDir: '/workingdir/Analysis/Control/'
HeatDir: '/workingdir/Analysis/Heatmap/'
AlnQCDir: '/workingdir/Analysis/Alignment_Statistics/'
CountQCDir: '/workingdir/Analysis/ReadCount_Statistics/'
ScatterDir: '/workingdir/Analysis/ReadCount_Scatterplots/'
HiLiteDir:
'/workingdir/Analysis/ReadCount_Scatterplots/Highlighted_Genes/'
CorrelDir: '/workingdir/Analysis/Replicate_Correlation/'
HiLiteDir2:
'/workingdir/Analysis/Replicate_Correlation/Highlighted_Genes'
EffDir: '/workingdir/Analysis/sgRNA_Efficacy/'
```

DepthDir: '/workingdir/Analysis/Read_Depth/'
SeqQCDir: '/workingdir/Analysis/Sequence_Quality/'
pvalDir: '/workingdir/Analysis/p-values/'
LogFileDir: '/workingdir/Analysis/Log_File/'
bw2Dir: '/usr/bin/'
CutAdaptDir: '/root/.local/bin/'
STARSDir: '/opt/PinAPL-Py/Scripts/STARS_mod/'

SanityScript: 'CheckCharacters'
IndexScript: 'BuildLibraryIndex'
LoaderScript: 'LoadDataSheet'
ReadDepthScript: 'PlotNumReads'
SeqQCScript: 'CheckSequenceQuality'
TrimScript: 'TrimReads'
AlignScript: 'AlignReads'
NormalizeScript: 'NormalizeReadCounts'
AverageCountsScript: 'AverageCounts'
StatsScript: 'AnalyzeReadCounts'
ControlScript: 'AnalyzeControl'
sgRNARankScript: 'FindHits'
GeneRankScript: 'RankGenes'
CombineScript: 'CombineGeneRanks'
ScatterScript: 'PlotCounts'
ReplicateScript: 'PlotReplicates'
ClusterScript: 'PlotHeatmap'

Supplementary Tables

Supplementary Table 1-3 and 5: Separate Excel files.

Supplementary Table 4: Summary of antibodies used in the study.

Target protein	Name	Manufacturer	Catalog #	Use	Dilution
AKT	Akt (pan) (40D4) Mouse mAb	Cell Signaling Technology	2920	Western Blot	1:1000
CD44	APC/Cy7 anti-mouse/human	Biolegend	103028	Flow cytometry	-
Cleaved caspase 3	Cleaved Caspase-3 (Asp175) (5A1E) Rabbit mAb	Cell Signaling Technology	9664	Western Blot	1:1000
Cleaved caspase 7	Cleaved Caspase-7 (Asp198) Antibody	Cell Signaling Technology	9491	Western Blot	1:1000
Cleaved caspase 8	Caspase-8 (1C12) Mouse mAb	Cell Signaling Technology	9746	Western Blot	1:1000
Cleaved caspase 9	Caspase-9 Antibody (Human Specific) #9502	Cell Signaling Technology	9502	Western Blot	1:1000
ERK1/2	p44/42 MAPK (Erk1/2) Antibody	Cell Signaling Technology	9102	Western Blot	1:1000
EWS-FLI1	FLI1	MyBiosource	MBS300723	Western Blot	1:1000
GAPDH	GAPDH (D16H11) XP	Cell Signaling Technology	5174	Western Blot	1:1000
P38	p38 MAPK Antibody	Cell Signaling Technology	9212	Western Blot	1:1000
p56/46 JNK	SAPK/JNK Antibody	Cell Signaling Technology	9258	Western Blot	1:1000
PARP	PARP Antibody	Cell Signaling Technology	9542	Western Blot	1:1000
PEG10	Anti-PEG10/EDR antibody [EPR20051]	Abcam	ab215035	Western Blot	1:1000
phospho-AKT	Phospho-Akt (Ser473) (D9E) XP Rabbit mAb	Cell Signaling Technology	4060	Western Blot	1:1000
phospho-ERK1/2	Phospho-p44/42 MAPK (Erk1/2) (Thr202/Tyr204)	Cell Signaling Technology	9101	Western Blot	1:1000
phospho-P38	Phospho-p38 MAPK (Thr180/Tyr182) Antibody	Cell Signaling	9211	Western Blot	1:1000

		Technology			
phospho-p56/46 JNK	Phospho-SAPK/JNK (Thr183/Tyr185) (81E11) Rabbit mAb	Cell Signaling Technology	4668	Western Blot	1:1000
phospho-SMAD2	Phospho-Smad2 (Ser465/467) (138D4) Rabbit mAb	Cell Signaling Technology	3108	Western Blot	1:1000
Mouse IgG	Anti-mouse IgG, HRP-linked Antibody	Cell Signaling Technology	7076	Western Blot	1:1000
Rabbit IgG	Anti-rabbit IgG, HRP-linked Antibody	Cell Signaling Technology	7074	Western Blot	1:1000
SGCE	SGCE Polyclonal Antibody	ThermoFisher Scientific	PA5-67363	Western Blot	1:1000
SMAD2	Smad2/3 (D7G7) XP Rabbit mAb	Cell Signaling Technology	8685	Western Blot	1:1000
TGFBR2	Recombinant Anti-TGF beta Receptor II antibody [EPR14673]	Abcam	ab184948	Western Blot	1:2500
β-tubulin	β-Tubulin (9F3) Rabbit mAb HRP Conjugate	Cell Signaling Technology	5346	Western Blot	1:1000
